# Increase of microRNA-210, Decrease of Raptor Gene Expression and Alteration of Mammalian Target of Rapamycin Regulated Proteins following Mithramycin Treatment of Human Erythroid Cells

**DOI:** 10.1371/journal.pone.0121567

**Published:** 2015-04-07

**Authors:** Nicoletta Bianchi, Alessia Finotti, Manuela Ferracin, Ilaria Lampronti, Cristina Zuccato, Giulia Breveglieri, Eleonora Brognara, Enrica Fabbri, Monica Borgatti, Massimo Negrini, Roberto Gambari

**Affiliations:** 1 Department of Life Sciences and Biotechnology, Ferrara University, Ferrara, Italy; 2 Laboratory for the Development of Pharmacological and Pharmacogenomic Therapy of Thalassaemia, Biotechnology Center, Ferrara University, Ferrara, Italy; 3 Department of Morphology, Surgery and Experimental Medicine, Ferrara University, Ferrara, Italy; 4 Laboratory for Technologies of Advanced Therapies (LTTA), Ferrara University, Ferrara, Italy; Southern Illinois University School of Medicine, UNITED STATES

## Abstract

Expression and regulation of microRNAs is an emerging issue in erythroid differentiation and globin gene expression in hemoglobin disorders. In the first part of this study microarray analysis was performed both in mithramycin-induced K562 cells and erythroid precursors from healthy subjects or β-thalassemia patients producing low or high levels of fetal hemoglobin. We demonstrated that: (a) microRNA-210 expression is higher in erythroid precursors from β-thalassemia patients with high production of fetal hemoglobin; (b) microRNA-210 increases as a consequence of mithramycin treatment of K562 cells and human erythroid progenitors both from healthy and β-thalassemia subjects; (c) this increase is associated with erythroid induction and elevated expression of γ-globin genes; (d) an anti-microRNA against microRNA-210 interferes with the mithramycin-induced changes of gene expression. In the second part of the study we have obtained convergent evidences suggesting raptor mRNA as a putative target of microRNA-210. Indeed, microRNA-210 binding sites of its 3’-UTR region were involved in expression and are targets of microRNA-210-mediated modulation in a luciferase reporter assays. Furthermore, (i) raptor mRNA and protein are down-regulated upon mithramycin-induction both in K562 cells and erythroid progenitors from healthy and β-thalassemia subjects. In addition, (ii) administration of anti-microRNA-210 to K562 cells decreased endogenous microRNA-210 and increased raptor mRNA and protein expression. Finally, (iii) treatment of K562 cells with premicroRNA-210 led to a decrease of raptor mRNA and protein. In conclusion, microRNA-210 and raptor are involved in mithramycin-mediated erythroid differentiation of K562 cells and participate to the fine-tuning and control of γ-globin gene expression in erythroid precursor cells.

## Introduction

The regulation of gene expression in normal and pathological conditions is finely tuned by several coordinated biological activities operating at the level of transcription [1], processing of primary transcripts [2], translation efficiency [3] and stability of the mature mRNAs [4]. In this context, the importance of microRNAs has been proposed and recently confirmed in several experimental systems [5–8].

MicroRNAs (miRs, microRNAs) are a family of small (19 to 25 nucleotide in length) noncoding RNAs that regulate gene expression by sequence-selective targeting of mRNAs [5,6], leading to a translational repression or mRNA degradation, depending on the degree of complementarity between microRNAs and the target sequences [7–9]. Since their discovery and first characterization, the number of microRNA sequences deposited in the miRBase databases is significantly growing [10]. On the other hand, considering that a single microRNA can target several mRNAs and a single mRNA might contain in its 3’-UTR sequence several signals for recognition by different microRNAs, it is calculated that at least 10–40% of human mRNAs are target of microRNAs [9]. Therefore, a great interest has been focused in recent years concentrated on the identification of validated targets of microRNAs. This specific field of microRNA research has confirmed that the complex networks constituted by microRNAs and mRNA targets coding for structural and regulatory proteins lead to the control of highly coordinated biological functions, such as differentiation, cell cycle and apoptosis [7–10]. More in detail, and considering the role of microRNAs, low expression of a given microRNA is expected to be linked with high levels of targets mRNAs; conversely, high expression of microRNAs is expected to be associated with low expression of the target mRNAs.

In this respect, increasing numbers of reports on the roles of microRNAs in erythropoiesis have been published [11–23]. For instance, Felli *et al*., identified microRNA-221 and microRNA-222 as being highly expressed in human cord blood derived hematopoietic CD34^+^ progenitor cells [11]. MicroRNA expression profiling was also performed by Choong *et al*. on *ex vivo* differentiating erythroid cultures derived from human umbilical cord blood (UCB) CD34^+^ cells and K562 cells, with the aim of identifying microRNAs involved in erythropoiesis [12]. MicroRNAs microRNA-15b, microRNA-16, microRNA-22, and microRNA-185 were found to have strong positive correlation with the appearance of erythroid surface antigens (CD71, CD36, and CD235a) and hemoglobin synthesis, while microRNA-28 displayed an inverse relationship with the expression of these markers. Other efforts aimed at defining erythroid-specific microRNAs were those published by Georgantas *et al*., who demonstrated microRNA-155 as a microRNA involved in the control of both myeloid and erythroid differentiation [13]. In conclusion, several microRNAs have been already proposed to play roles in normal hematopoiesis [14–23].

The aim of the present study was to compare the expression of microRNAs in two different complementary cell systems, human leukemic K562 cells induced to γ-globin gene expression by the fetal hemoglobin (HbF) inducer mithramycin (MTH) [24] and erythroid progenitor cells (ErPCs) isolated from the peripheral blood of healthy subjects and β-thalassemia patients [25]. To this aim, microarray analysis was performed employing (a) RNA isolated from healthy subjects, β-thalassemia patients expressing low levels of HbF, and β-thalassemia patients exhibiting a hereditary persistence of fetal hemoglobin (HPFH)-like phenotype, expressing almost exclusively HbF and (b) RNA from uninduced control and MTH-induced K562 cells [26, 27]. A second objective of this study was to identify possible mRNA targets of the most relevant microRNA(s) with particular attention to a possible link between microRNA expression and high production of HbF. To this aim, the effects of anti-microRNAs [28–29] and premicroRNA modulated silencing of the possible mRNAs targets studied [30].

## Materials and Methods

### Human cell lines and culture conditions

Human leukemia K562 cells [31] were grown in RPMI 1640 medium (Sigma, St.Louis, MO, USA) supplemented with 10% fetal bovine serum (FBS) (Biowest, Nuaillé, France), 50 U/ml penicillin and 50 μg/ml streptomycin in a humidified atmosphere (5% CO_2_/air) [32]. Mithramycin (MTH) and rapamycin (RAPA) were from Sigma (St.Louis, MO, USA). Stock solutions of MTH (100 μM) and RAPA (500 μM) were stored at -20°C in the dark and resuspended before the use. Treatment of K562 cells with erythroid differentiation inducers was carried out by adding the appropriate drug concentrations at the beginning of the cultures (cells were seeded at 30000/ml). The optimal MTH concentration was found to be 20–30 nM, depending of the batch used and was verified before each set of experiments by checking the effects on cell growth and differentiation, with the objective to use, when appropriate, experimental conditions in which MTH exerted similar general effects. The medium was not changed during the induction period. We evaluated antiproliferative effects of the compound using a Z1 Coulter Counter (Coulter Electronics, Hialeah, FL, USA). Erythroid differentiated K562 cells containing hemoglobin were detected by specific reaction with a benzidine/hydrogen peroxide solution as reported elsewhere [32]. The final concentration of benzidine was 0.2% in 5 M glacial acetic acid and 10% H_2_O_2_ [32].

### Transfection procedures using anti-microRNAs, premicroRNAs and siRNAs

The transfection protocol with anti-microRNAs, premicroRNAs and siRNAs was reported from Ambion (Applied Biosystems, Foster City, CA, USA) and 30000 K562 cell/ml were seeded in 24-well plates. MTH was administrated 6 hours after the first anti-microRNAs treatment; repeated treatments with 200 nM anti-microRNAs and premicroRNAs were performed for 5 days consecutively using siPort NeoFX transfection reagent. Transfection with siRNAs was carried out employing 200 nM Silencer GAPDH siRNA or pre-designed siRNAs targeting raptor mRNA (catalog number ID s33214) purchased from Ambion (Applied Biosystems, Foster City, CA, USA). Gene-silencing was obtained by administration of siRNA molecules for two days. The Cells-to-cDNA II Kit (Applied Biosystems, Foster City, CA, USA) was used to evaluate mRNA content. 25 μl of lysis buffer were added to cell pellets and 6 μl employed for production of the cDNAs to be analysed by real-time PCR.

### Ethics Statement

The use of human material was approved by the Ethics Committee of Ferrara's District, document number 06/2013, approved on 20th of June 2013. All samples of peripheral blood have been obtained after receiving written informed consent from patients and healthy donors or their legal representatives. Copies of the consents have been collected for archiving by the “Day Hospital Talassemici”, Divisione Pediatrica of Hospital S. Anna, Ferrara, Italy. The K562 cell line used in this study has been object of several previously published reports [24, 27, 31–32].

### Patients and erythroid precursor cultures

The microRNA profiling experiments were performed on ErPCs isolated from healthy subjects (d1-US and d2-US), β-thalassemia patients expressing very low levels of HbF (range 1.11–2.5%) and needing frequent transfusions associated with chelation therapy (ThFe30, ThFe42, ThFe52) and patients presenting HPFH-like phenotype (ThFe37 and Th17) with about 40% HbF and over 88% HbF, respectively. In particular, Th17 patient is a 13 years old girl with a novel thalassemia mutation, a single A nucleotide insertion within the exon 1, at codon 18, of the β-globin gene associated with a deletion of the δβ-globin gene region, who has never been transfused, despite the fact that she does not produce any detecs level of adult hemoglobin (HbA) [26]. Written informed consent was obtained from each patient and the samples of peripheral blood were collected just before the transfusion treatment.

The Ficoll-Hypaque density gradient centrifugation was used to purify peripheral blood mononuclear cells. After isolation, the mononuclear cell layer was washed three times by adding 1x phosphate-buffered saline (PBS) solution. The pellet was then resuspended in α-minimal essential medium supplemented with 10% FBS (Celbio, Milano, Italy), 1 μg/ml cyclosporine A (Sigma Aldrich St. Louis, Missouri, USA) and 10% conditioned medium obtained from supernatant of the 5637 bladder carcinoma cell line culture. These mononuclear cell suspension was grown in these culture conditions for seven days at 37°C, under an atmosphere of 5% CO_2_ in air, with extra humidity (phase I culture) [33]. The nonadherent cells were harvested from the flask, washed in 1x PBS, and then grown in α-medium, 30% FBS, 1% deionized bovine serum albumin (BSA), 10^-5^ M β-mercaptoethanol, 1.5 mM L-glutamine, 10^-6^ M dexamethasone, and 1 U/ml human recombinant erythropoietin (EPO) (Tebu-bio, Magenta, Italy) and stem cell factor (SCF) (Inalco, Milano, Italy) (phase II culture) [33]. After seven days of cell culture phase II, the cells were treated for additional four days in the presence of the erythroid inducer MTH [34].

### Fluorescence-activated cell sorting (FACS) analysis

The erythroid differentiation status of the ErPC cultures was investigated studying Transferrin Receptor (TR) and Glycophorin A (GYPA) expression by FACS analysis using anti-human CD71 FITC-conjugated antibody (ImmunoTools GmbH, Friesoythe, Germany) and polyclonal anti-human Glycophorin A antibody (Pierce Biotechnology, USA). To this aim, 10 μl of anti-human CD71 FITC-conjugated and 1 μl of anti-human GYPA antibodies were added to freshly isolated cells on ice in 100 μl 1x PBS and 0.1% fetal calf serum (FCS) for 30 min. Then, when appropriate, rabbit IgG-heavy and light chain cross-adsorbed R-Phycoerythrin conjugate antibody (Bethyl, Temaricerche, Italy) was added (1:50) to PBS-washed cells and a further incubation in ice (1x PBS, 0.1% FBS) was carried out for 30 min. Finally, cells were washed in 1x PBS and analyzed using the BD FACScan system (Becton, Dickinson & Company, Italy).

### RNA extraction

Cells were isolated by centrifugation at 1500 rpm for 10 min at 4°C, washed in PBS, lysed in Tri-reagent (Sigma Aldrich, St. Louis, MO, USA), according to the manufacturer’s instructions. The isolated RNA was washed once with cold 75% ethanol, dried and resuspended in diethylpyrocarbonate-treated water before use.

### Human microRNAs/mRNA expression detection by microarray-based analyses

The microRNAs profiling was performed using the Agilent Human microRNA microarray v.2 (#G4470B, Agilent Technologies, Palo Alto, CA, USA). Labelled RNA was hybridized according to the manufacturer’s procedure. This chip, consisting of 60-mer DNA probes, contains 15000 features, which represent 723 microRNAs, sourced from the Sanger miRBase database (Release 10.1). The global mRNA expression was detected using the Agilent whole human genome oligo microarray (#G4112F, Agilent Technologies). This microarray consists of 60-mer DNA probes synthesized in situ, which represent 41000 unique human transcripts. About 500 ng of total RNA were employed in each experiment. RNA labeling and hybridization were performed in accordance to manufacturer’s indications. Images at 5 μm resolution were generated by Agilent scanner and the raw-data were obtained by Feature Extraction 10.5 software (Agilent Technologies, Palo Alto, CA, USA). Microarray results were analyzed using the GeneSpring GX 10 software (Agilent Technologies, Palo Alto, CA, USA). Data transformation was applied to set all negative raw values at 1.0, followed by a normalization on 75^th^ percentile. A filter on low gene expression was used to keep only the probes expressed in at least one sample (flagged as Marginal or Present). Differentially expressed genes were selected by using fold-change analysis and, when applicable, the ANOVA (analysis of variance) statistic (p<0.05). Differentially expressed genes were employed for Cluster Analysis of samples using the Pearson correlation as a measure of similarity.

### Bioinformatics and pathway analysis

Different bioinformatics tools used to identify putative target genes predicted by at least two algorithms among TargetScanS 4.2 (http://www.targetscan.org/), MiRanda 4.0 (http://www.ebi.ac.uk/enright-srv/microcosm/htdocs/targets/v5/) and PicTar (http://www.pictar.org/). The microRNA database provides further available information (http://www.microrna.org/). The Pathway Enrichment Analysis was performed using GeneSpring GX tools, based on KEGG, BioCarta, Biopax, Cellmap and NCI-Nature databases.

### Reverse Transcription and quantitative real-time PCR (RT-qPCR)

The reagents, primers and probes for microRNAs quantification by real-time RT-PCR were obtained from Applied Biosystems (Foster City, CA, USA). Reverse transcription (RT) reactions were performed using TaqMan MicroRNA Reverse Transcription Kit (Applied Biosystems, Foster City, CA, USA) and real-time PCR were performed according to manufacturer’s protocols. 20 nanograms per sample was used for the assays. All RT reactions, including no-template controls and RT-minus controls, was performed in duplicate using the 7700 Sequence Detection System version 1.7 (Applied Biosystems, Foster City, CA, USA). Relative expression was calculated using the comparative cycle threshold method and as reference gene hsa-microRNA-let-7c microRNA and U6 snRNA were used to normalize all RNA samples, because they are equally expressed in the assayed samples when analyzed by microRNAs-profiling and RT-qPCR analysis as previously reported [27]. For gene expression analysis 500 ng of total RNA were reverse transcribed using random hexamers. RT-qPCR assay was carried out using gene-specific double fluorescently labeled probes. The nucleotide sequences used for real-time qPCR analysis of α-, β- and γ-globin mRNAs are here reported: α-globin forward primer, 5’-CAC GCG CAC AAG CTT CG-3’, α-globin reverse primer, 5’-AGG GTC ACC AGC AGG CAG T-3’, α-globin probe, 5’-FAM-TGG ACC CGG TCA ACT TCA AGC TCC T-TAMRA-3’, β-globin forward primer, 5’- CAA GAA AGT GCT CGG TGC CT -3’, β-globin reverse primer, 5’-GCA AAG GTG CCC TTG AGG T-3’, β-globin probe, 5’-FAM-TAG TGA TGG CCT GGC TCA CCT GGA C-TAMRA-3’, γ-globin forward primer, 5’-TGG CAA GAA GGT GCT GAC TTC-3’, γ-globin reverse primer, 5’-TCA CTC AGC TGG GCA AAG G-3’, γ-globin probe, 5’-FAM-TGG GAG ATG CCA TAA AGC ACC TGG-TAMRA-3’ [25, 32, 35]. The primers and probes used to assay the expression of raptor mRNA (Assay ID Hs00977502_m1), FANK1 (fibronectin type III and ankyrin repeat domains 1) (Assay ID Hs01113524_m1), CYB5R2 (cytochrome b5 reductase 2) (Assay ID Hs00212055_m1) and others genes reported were purchased from Applied Biosystems (Applied Biosystems, Foster City, CA, USA). Relative expression was calculated using the comparative cycle threshold (CT) method and the endogenous control human 18S rRNA as reference gene.

### High Performance Liquid Chromatography (HPLC)

K562 cells were harvested, washed once with PBS and the pellets were lysed in lysis buffer (sodium dodecyl sulphate 0.01%). After incubation on ice for 15 min, and spinning for 5 min at 14000 rpm in a microcentrifuge, the supernatant was collected and injected. Hb proteins present in the lysates were separated by cation-exchange HPLC [25, 35], using a Beckman Coulter instrument System Gold 126 Solvent Module-166 Detector. Hemoglobins were separated using a PolyLC (Columbia, MD, USA) PolyCAT-A model (35 mmx4.6 mm) column; samples were eluted in a solvent gradient using aqueous sodium chloride-BisTris-KCN buffers and detection was performed at 415 nm. The standard controls were the purified HbA (SIGMA, St Louis, MO, USA) and HbF (Alpha Wassermann, Milano, Italy).

### Extract preparation

Treated or untreated K562 cells (2x10^5^) were washed three times with cold 1x PBS and centrifuged at 1200 rpm for 10 min at 4°C. Then, cellular pellets were resuspended in 50 μl cold water, frozen by dry ice for 5 min and vortexed for 10 s. This step was repeated four times consecutively. Samples were finally centrifuged at 14000 rpm for 20 s and the supernatant cytoplasmic fractions were collected and immediately frozen at -80°C. Protein concentration was determined according to the Bradford method [36].

### Western blotting

For Western blotting analyses 10 μg of cytoplasmic extracts were denatured for 5 min at 98°C in 1x sodium dodecyl sulfate (SDS) sample buffer (62.5 mM Tris-HCl pH 6.8, 2% SDS, 50 mM Dithiotreithol (DTT), 0.01% bromophenol blue, 10% glycerol) and subjected to SDS/polyacrylamide gel electrophoresis (SDS/PAGE) (8% polyacrylamide). Proteins transfer to 20 μm nitrocellulose membrane (Pierce, Euroclone S.p.A., Pero, Milano, Italy) was performed overnight at 360 mA and 4°C in 25 mM Tris, 192 mM Glycine, 5% methanol. After prestaining with a Ponceau S Solution (Sigma, St.Louis, MO, USA), the membrane was blocked with 5% Milk and 1x Tris-buffered saline and Tween-20 0.1% (TBS/T) for 1 hour at room temperature, washed three times and left with primary rabbit monoclonal antibody (1:1000) in 5% BSA and 1x TBS/T overnight at 4°C. All used monoclonal antibodies (p70, Phospho-p70 Thr389, mTOR (mammalian target of rapamycin), Phospho-S6 Ribosomal Protein Ser235/236, raptor) were purchased from Cell Signaling (Euroclone S.p.A., Pero, MI, Italy). Then, the membrane was washed three times, incubated for 2 hours at room temperature with appropriate anti-rabbit IgG HRP-linked antibody diluted 1:2000 in 5% Milk and 1x TBS/T and HRP-linked anti-biotin antibody diluted 1:1000 (to detect biotinylated protein marker) (Cell Signaling, Euroclone S.p.A., Pero, MI, Italy). Finally, the membrane was incubated for 5 min at room temperature with LumiGLO (0.5 ml 20x LumiGLO, 0.5 ml 20x Peroxide and 9.0 ml Milli-Q water) (Cell Signaling, Euroclone S.p.A., Pero, MI, Italy) and exposed to X-ray film (Pierce, Euroclone S.p.A., Pero, MI, Italy). When necessary, after a stripping procedure using the Restore Western Blot Stripping Buffer (Pierce, Euroclone S.p.A., Pero, MI, Italy) membranes were re-probed with primary and secondary antibodies. X-ray films for chemiluminescent blots were analyzed by Gel Doc 2000 (Bio-Rad Laboratoires, MI, Italy) using Quantity One program to elaborate the intensity data of our specific target protein. Ponceau S staining was used as loading control (S1 Fig), together with other markers were taken as reference tools (for example mTOR and p70).

### Cloning of raptor microRNA-210 target sites in the pmiRGLO vector and luciferase assay

The protocol reported from Promega Corporation (WI, USA) was used for the cloning of raptor microRNA-210 target sites (site1: 5’-AAA CTA GCG GCC GCT CAC TGA GCA GGA AGC GCA CAG TCT AG-3’; site2: 5’-AAA CTA GCG GCC GCG AAG CCC AGC TCC ACC CGC ACA CTC TAG-3’) and mutated target sites (5’-AAA CTA GCG GCC GCT CAC TGA GCA GGC AGA TCA ACG TCT AG-3’; 5’-AAA CTA GCG GCC GCG AAT CGC AGA TCC TCC CTC GCA CTC TAG-3’). These oligonucleotide sequences contain 5’-PmeI, 3’-XbaI, and NotI (for clonal selection) restriction sites. The names of the generated clones were raptor-210-site1-pmiRGLO, raptor-210-site2-pmiRGLO, MUT-raptor-210-site1-pmiRGLO and MUT-raptor-210-site2-pmiRGLO. For the luciferase assay, 50 ng of plasmid were used to transfect 500000 K562 cells in 100 μl of MiRus Bio Ingenio Electroporation Solution (Temaricerca, Bologna, Italy). In some experiments, as reported in the Results section, 400 nM premicroRNA-210 was also co-tranfected. The cell/DNA suspension was transferred into 0.2 cm Ingenio cuvettes (Temaricerca, Bologna, Italy). The appropriate Nucleofector Program (T-016 for Nucleofector I Device) was selected and 500 μl of the culture medium was immediately added to the cuvette, gently transferred to 12-well plates with 2 ml of supplemented Optimem Gibco medium (Life technologies, Monza, Italy) and pre-incubated in a humidified 37°C/100% air incubator without CO_2_. After 24 hours, 10 μl of the lysates were analyzed with the Dual Luciferase assay (Promega Corporation, WI, USA) to detect firefly luciferase and renilla luciferase activity.

### Statistical analysis

All the data were normally distributed and presented as mean±S.D. Statistical differences between groups were compared using one-way ANOVA (ANalyses Of VAriance between groups) software. P values were obtained using the Paired t test of the GraphPad Prism Software. Statistical differences were considered significant when p<0.05 (*), highly significant when p<0.01 (**).

## Results

### Characterization of the differentiation status in of ErPC cultures and its correlation with hemoglobin production

**Fig 1:**
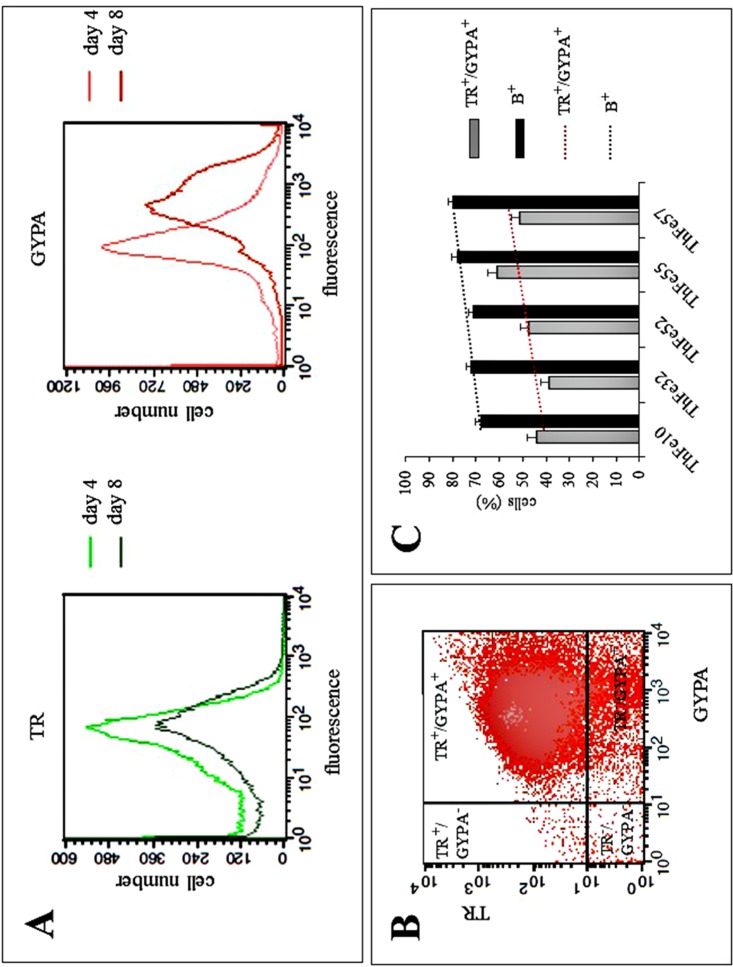
Expression of transferrin receptor (TR) and Glycophorin A (GYPA) in ErPCs analyzed by flow cytometry. (A) FACS analysis of TR (green) and GYPA (red) antibody binding to ErPCs isolated after day 4 and day 8 of cell culture. (B) Relationship between TR and GYPA expression in day 4 ErPCs cultures. (C) Comparison of TR+/GYPA+ and B+ (benzidine-staining positive) cells in ErPCs from different β-thalassemic patients (n = 5, p<0.05).

The expression of transferrin receptor (TR) and glycophorin A (GYPA) surface markers was firstly investigated in the ErPC phase II cultures obtained from β-thalassemia patients, in order to define their correlation with Hb production. In Fig 1A, a representative example of flow cytometry analysis is shown relative to ErPC samples isolated after 4 and 8 days of cell culture. The results obtained clearly indicate an increase of GYPA expression from day 4 to day 8 of cell culture, when most of the cells are positive to the benzidine-staining, indicating high Hb production. On the contrary, the intensity of the expression of TR does not increase, confirming that full activation of TR functions is an early marker of ErPC differentiation, while fully activation of GYPA occurs at later stages. Remarkably, the proportion of TRF^+^ cells and GYPA^+^ cells is very high both at day 4 and day 8 of cell culture (>80%). Fig 1B reports a bi-parametric FACS analysis showing that even at day 4 most of the ErPCs are both TR^+^ and GYPA^+^. The representative example reported in Fig 1B was confirmed in 10 independent experiments. Fig 1C shows that a correlation does exist between the proportion of ErPCs expressing TR and GYPA erythroid differentiation markers and their Hb content in samples from 5 β-thalassemia patients. These analyses allow concluding that EPO-cultured ErPCs, in addition to Hb production, exhibit high expression of erythroid markers.

### MicroRNA profile in ErPCs and K562 cells: microRNA-210 is up-regulated in ErPCs from β-thalassemia patients expressing high HbF levels and in K562 cells induced to erythroid differentiation by MTH

**Fig 2:**
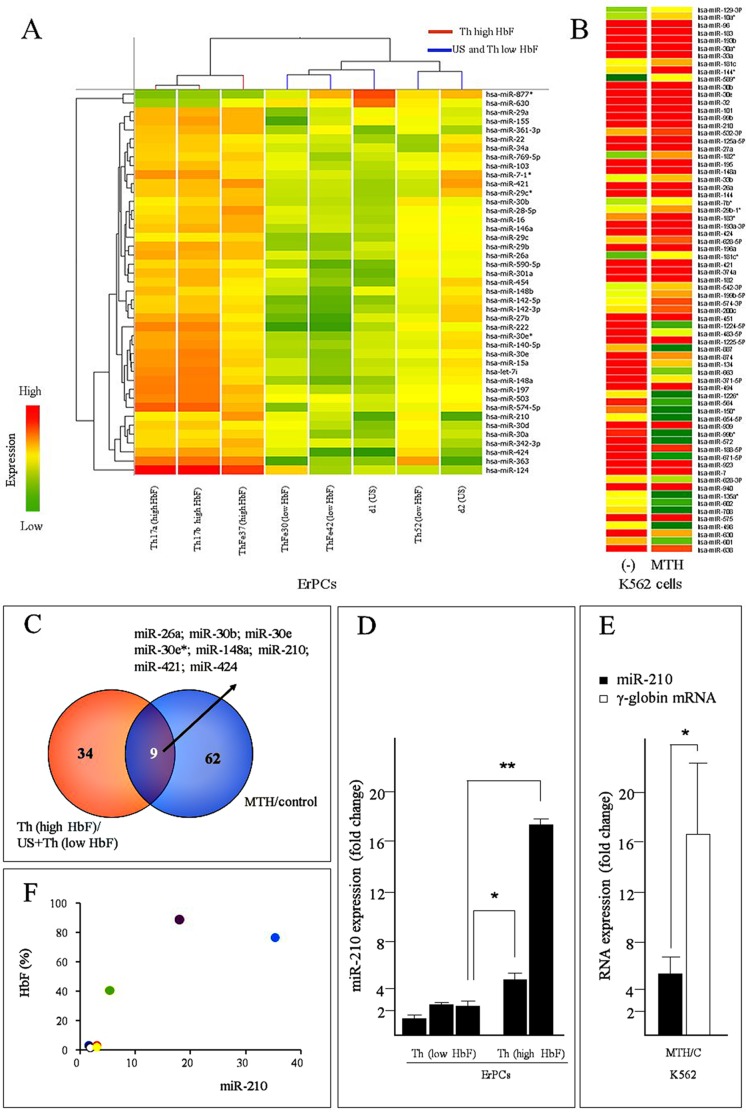
MicroRNAs modulated in ErPCs from β-thalassemia patients and healthy donors and in K562 cells induced to erythroid differentiation by MTH. (A) Hierarchical clustering of ErPC samples from β-thalassemia patients and healthy donors (unaffected subjects, US). These samples were clustered according to the expression profile of the 43 microRNAs differentially expressed in samples from β-thalassemia patients with high HbF (Th17a, Th17b, ThFe37), with low HbF (ThFe30, ThFe42, ThFe52) and healthy donors (d1-US, d2-US). Different sample subtypes are indicated with color bars (red, high HbF; blue, US and low HbF). The differential expression of microRNAs can be easily followed by a color-code approach, indicating with green low levels, and with red high levels of microRNA content. (B) Heat map of the 71 microRNAs up- or down-regulated in K562 cells following MTH treatment. (-) = K562 cells untreated control. (C) Common microRNAs modulated in Th (high HbF) *vs*. Th (low HbF) plus US samples and in MTH-induced K562 *vs*. uninduced cells. (D) MicroRNA-210 expression analyzed by RT-qPCR in RNA samples from two Th (high HbF) ErPCs and three Th (low HbF) ErPCs. The data are represented as fold change with respect to ErPCs from healthy subjects (* = p<0.05; ** = p<0.01). (E) Changes of microR-210 (black) and γ-globin mRNA (white) expression in MTH-induced K562 cells in five independent experiments (* = p<0.05). The data represent fold change with respect to control untreated K562 cells. MicroRNA-let-7c (equally expressed in the samples) and 18S were used as internal reference gene sequences to normalize RNA input. (F) Correlation between microRNA-210 content in ®-thalassemia patient ErPCs (analyzed by RT-qPCR with respect to healthy subjects) and %HbF (analyzed by HPLC) in peripheral blood.

For the microarray-based screening we used the Agilent microRNA microarray, which contains 748 microRNA probes. The first screening was performed using RNA from ErPCs isolated from three different classes of subjects: unaffected healthy donors (d1-US, d2-US), β-thalassemia patients with low HbF levels (HbF<2%: ThFe30, ThFe42, ThFe52) and β-thalassemia patients with high HbF levels (HbF>40%: two samples from Th17 and one from ThFe37) [26]. Biochemical features (HbF content and genotype) of ErPCs of Th17 has been already reported elsewhere [26, 27]. As expected, erythroid cells derived from healthy donors accumulate α-globin and β-globin mRNAs and produce high levels of HbA and very low levels of HbF (data not shown). Erythroid cells from ThFe30, ThFe42 and ThFe52 accumulate very low levels of β-globin mRNA, low levels of γ-globin mRNA and levels of α-globin mRNA comparable to the healthy donors; accordingly, erythroid cells from these patients express low levels of HbF (1.71%±0.61, n = 3). As previously published by our group, Th17 erythroid cells accumulate, in addition to the α-globin, very high levels of γ-globin mRNA, HbF being the major hemoglobin produced by this HPFH patient (HbF>85%±5.5, n = 5) [26]. The ErPCs from the second HPFH patient (ThFe37) express α-globin, high levels of γ-globin mRNA and high levels of HbF (40.06%±0.43, n = 3) (data not shown). All the cellular cultures used in the microRNA profiling reported in Fig 2A displayed more than 80% erythroid differentiated cells, as judged by benzidine-staining (data not shown). The complete list of microRNAs, whose expression is modified (using 2.00 as fold change value), is reported in S1 Table, which enlists the top 43 differentially expressed microRNAs originating from the comparison of the mean of the data relative to the microRNA-profiles of Th (high HbF) *vs*. US plus Th (low HbF).

The second screening was performed on RNA isolated from either untreated K562 cells or K562 cells treated for 4 days with 20 nM MTH. This relatively low concentration of MTH was chosen in order to avoid the identification of microRNAs whose expression is altered in association with effects on cell growth, rather than activation of erythroid functions. After comparison of the analysis of both uninduced and MTH-induced K562 cells, the expression of 71 microRNAs was found to be up- or down-regulated using as threshold a 1.75 fold change value [27]. The 1.75 fold change was chosen in order to obtain a number of upregulated microRNAs comparable to that obtained when we used ErPCs as biological samples. The Fig 2B reports the heat map of these microRNA analyses. The complete list of microRNAs whose expression is changed following MTH induction of K562 cells is reported in S2 Table, which contains the normalized expression values.

In order to identify microRNAs whose expression could be linked to high production of HbF, we compared the list of S1 Table (including the 43 microRNAs differentially expressed in ErPCs producing high HbF) with those modulated in MTH-induced K562 cells (reported in S2 Table). Interestingly, 9 microRNAs were found in common, 8 of them being up regulated (Fig 2C). Among these microRNAs, microRNA-210 on one hand is the most expressed in high-HbF samples and, on the other, exhibits a high absolute fold change value. Therefore, we pointed our attention on microRNA-210. In addition, this microRNA has been previously described to be up regulated during differentiation [23] and high HbF expression [27]. Furthermore, microRNA-210 is known as firmly associated to hypoxia; this is in our opinion of interest, since hypoxia has been demonstrated to be associated to activation of the erythroid program leading to selective expression of fetal globin genes and HbF production in erythroid cells [37–40].

Fig 2D shows the validation by RT-qPCR of microRNA-210 expression data obtained by the microRNA-profiling. The results indicate a slight increase of microRNA-210 in ErPCs from β-thalassemia patients, in comparison to ErPCs from healthy donors. In agreement with the microarray data, β-thalassemia ErPCs expressing elevated HbF levels exhibit an even higher content of microRNA-210.

Furthermore, the increase of microRNA-210 and γ-globin mRNA content in MTH-treated K562 cells (Fig 2E) was confirmed in five independent experiments, in agreement with data elsewhere reported (Paired t test, p = 0.0123) [27] and microarray results (Fig 2B and S2 Table), suggesting that the increase of microRNA-210 is a reproducible feature and could be associated to γ-globin gene expression.

Finally, we observed a significant correlation between microRNA-210 content and HbF in ErPCs from β-thalassemia patients (α = 0.05%, two-tailed = 0.0176 and R^2^ = 0.7082) (data elaborated with GraphPad program) (Fig 2F).

Altogether, the data shown in Fig 2 (panels A, E and F) encouraged us to follow the hypothesis of a possible link between expression of microRNA-210 and HbF levels in our experimental model systems.

### Anti-microRNA against microRNA-210 prevents MTH-mediated gene expression changes

**Fig 3:**
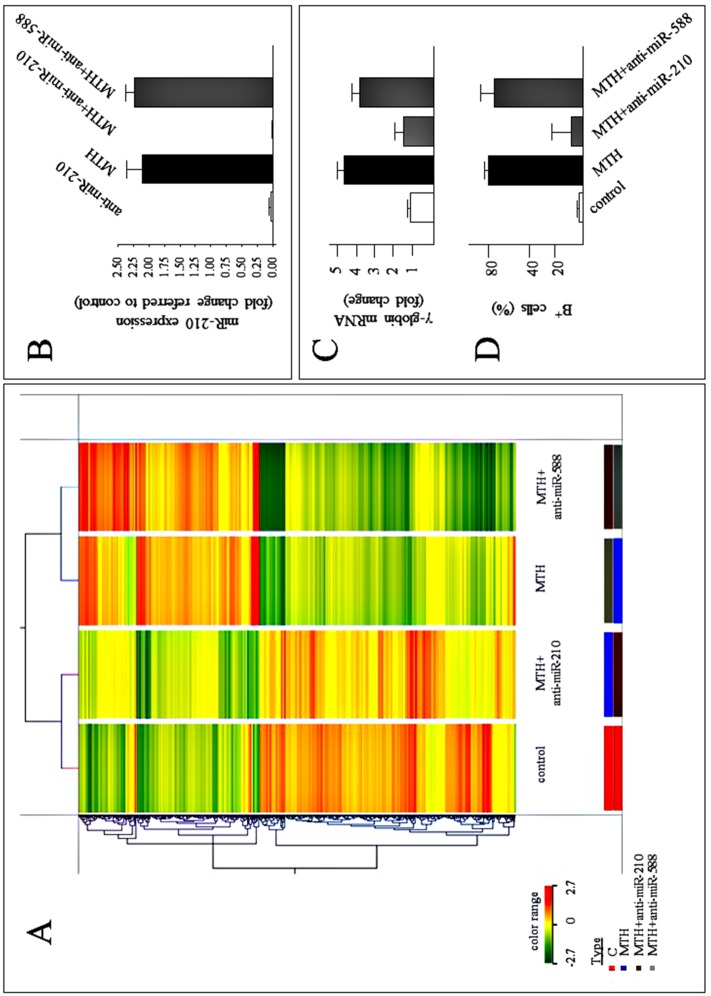
Modulation of gene expression by anti-microRNA-210 in K562 cells induced to erythroid differentiation by MTH. (A) Heat map representation of 748 mRNAs clustered according to the expression profile. Cell cultures were carried out for six days. The samples analyzed were obtained from untreated K562 cells and K562 cells treated with MTH (30 nM) + anti-microRNA-210, MTH, or MTH + anti-microRNA-588 (taken as anti-microRNA-control). (B) RT-qPCRs of microRNA-210 in K562 cells treated with anti-microRNA-210 (white histogram), K562 cells induced with 30 nM MTH in the absence (black histogram) and in the presence of anti-microRNA-210 or anti-microRNA-588 (grey histograms). The cells were seeded at 10000 cells/500 μl and 30 nM MTH added at the first day of culture after anti-microRNA treatment. The data depicted represent relative fold increase with respect to untreated cells (mean±S.D. from four independent experiments). MicroRNA-let-7c was used as internal reference gene to normalize RNA input. (C) RT-qPCRs of γ-globin mRNA and (D) proportion of benzidine-positive cells (containing Hb) determined in untreated K562 cells (white histogram) and K562 cells treated with 30 nM MTH in the absence (black histogram) and in the presence of anti-microRNAs (grey histograms: anti-microRNA-210 or anti-microRNA-588, as indicated).

To further investigate the role of microRNAs in MTH-treatment, the gene expression profiles of K562 cells treated with 30 nM MTH and MTH plus anti-microRNA-210 or anti-microRNA-588 were examined. As previously described, microRNA-210 is up regulated after MTH-treatment, while microRNA-588 is expressed at very low levels in K562 cells without major changes during MTH-treatment (data not shown) and was considered as an internal negative control of the experiment. As expected, the results of this experiment demonstrate that MTH induces significant differences in the transcriptomic pattern (Fig 3A). We found only minor changes after treatment of K562 cells with MTH plus anti-microRNA-588, while treatment with MTH plus anti-microRNA-210 displays a microarray profile that is more similar to control cells than MTH-induced cells (Fig 3A). Overall, after comparison of the data obtained, 748 mRNAs were found to be modulated in MTH-treated cells (using 2.00 as fold change). From this gene expression profile, 358 genes among 748 mRNAs were found down-regulated by MTH and selectively induced by microRNA-210 inhibition with anti-microRNA-210.

Fig 3B reports the expression of microRNA-210 validated by RT-qPCR in samples treated with anti-microRNA-210. The results demonstrate that microRNA-210 expression is inhibited following treatment with the anti-microRNA-210 of MTH-treated and untreated K562 cells, but not when anti-microRNA-588 was used (Fig 3B). In addition, administration of anti-microRNA-210 (but not of anti-microRNA-588) prevents MTH-mediated γ-globin mRNA accumulation (Fig 3C). This is in agreement with the data shown in Fig 3D, indicating that the proportion of benzidine-positive cells (which is about 80% in MTH-treated K562 cells) decreases to about 5% in K562 cells treated with MTH plus anti-microRNA-210. Despite few differences in the heat map profile (Fig 3A), we found evidence suggesting that in the presence of anti-microRNA-210 the MTH-mediated erythroid induction is not fully operating.

**Fig 4:**
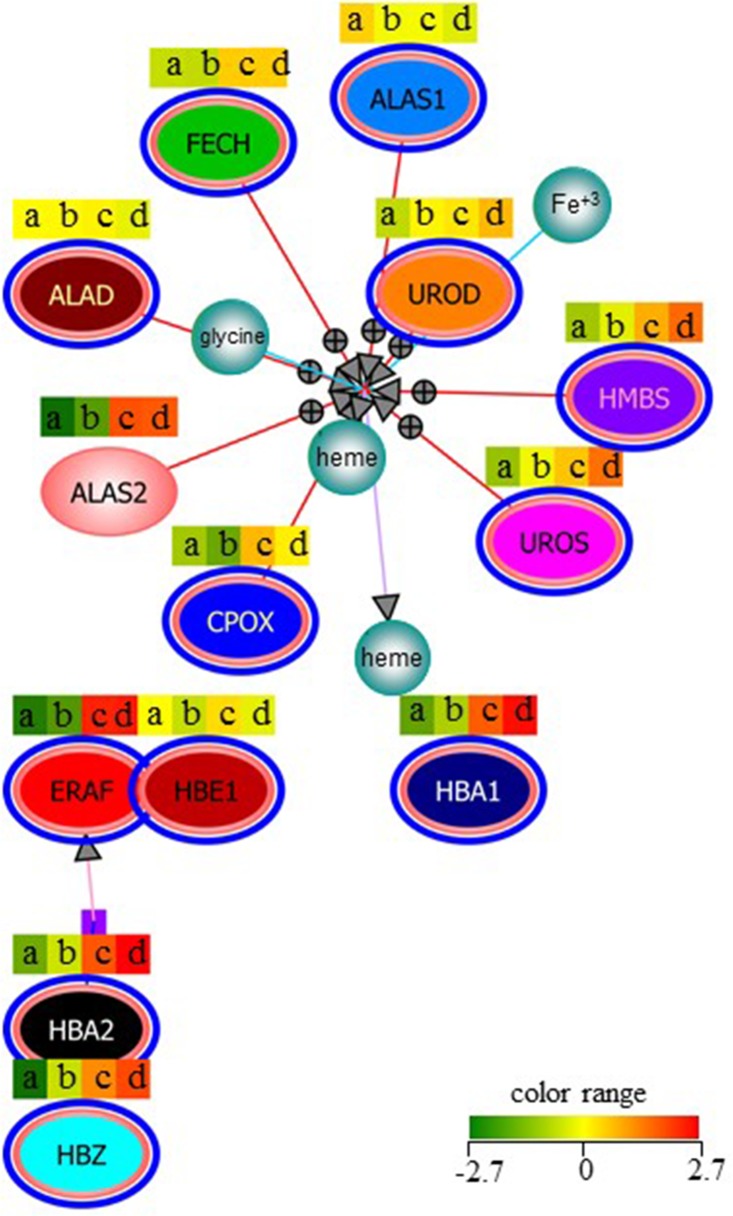
Modulated genes following anti-microRNA-210 treatment analyzed by gene-profiling and involved in heme biosynthetic process and hemoglobin pathway. The samples analyzed were obtained respectively from untreated K562 cells (a) or K562 cells treated with MTH (30 nM) + anti-microRNA-210 (b), MTH (c), or MTH + anti-microRNA-588 (d) (p<0.05). The differential expression of microRNAs can be easily followed by a color-bar approach indicating low levels (green) and high levels (red) of mRNA content.

In order to obtain further information supporting an effect of anti-microRNA-210 on the erythroid pattern and other erythroid-related genes, a computer-based analysis was performed on the pathways enriched in our list of modulated genes, showing that the hemoglobins chaperone is the most significant (S3 Table). In Table 1 the erythroid specific genes analyzed are enlisted and comprise hemoglobins, genes involved in heme biosynthetic process and other erythroid differentiation markers. The data shown in Table 1 and depicted in Fig 4 support the concept that treatment with anti-microRNA-210, but not with anti-microRNA-588, suppresses MTH-induced increases of the expression of these genes. These results strongly sustain the hypothesis that microRNA-210 (and its up regulation) is deeply involved in erythroid differentiation.

**Table 1:** Fold increase of the genes involved in the significant pathways obtained by gene expression profiling.

Hemoglobins chaperon^1^	MTH 30nM/C	MTH 30nM+anti-microRNA-210/C	MTH 30nM+anti-microRNA-588/C
HBA1	6.13	1.04	5.99
HBA2	7.12	1.09	6.76
HBE1	1.60	0.97	1.36
HBZ	5.30	1.47	5.30
ERAF	18.24	1.62	14.30
CPOX	2.74	1.10	2.66
HMBS	2.74	1.12	2.89
UROS	2.56	1.32	2.47
UROD	1.57	1.12	1.54
FECH	2.09	0.78	1.82
**Erythroid differentiation Markers** ^**2**^	**MTH 30nM/C**	**MTH 30nM+anti-microRNA-210/C**	**MTH 30nM+anti-microRNA-588/C**
NFE2L3	3.08	1.00	2.99
EPB49	3.58	1.13	3.40
SLC4A1	39.72	1.23	29.53

The values referred to untreated K562 cells control.

^1^. HBA1 (hemoglobin alpha 1), HBA2 (hemoglobin alpha 2), HBE1 (hemoglobin epsilon 1), HBZ (hemoglobin zeta), ERAF (erythroid associated factor), CPOX (coproporphyrinogen oxidase), HMBS (hydroxymethylbilane synthase), UROS (uroporphyrinogen III synthase), UROD (uroporphyrinogen decarboxylase), FECH (ferrochelatase)

^2^. NFE2L3 nuclear factor erythroid-derived 2-like 3), EPB49 (erythrocyte membrane protein band 4.9), SLC4A1 (solute carrier family 4, anion exchanger, member 1 erythrocyte membrane protein band 3, Diego blood group).

**Fig 5:**
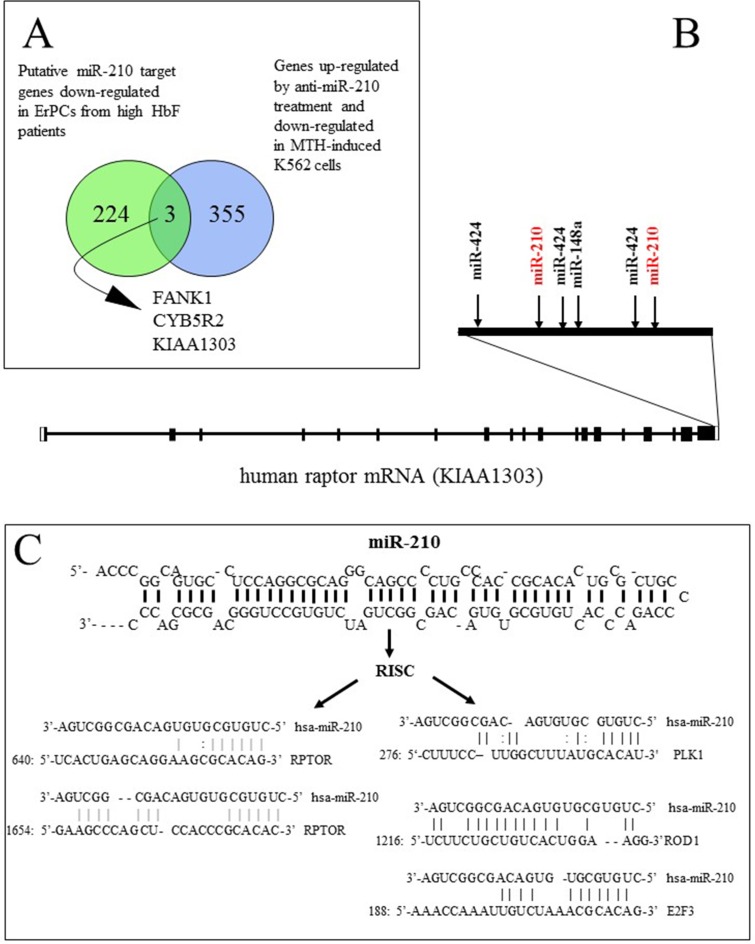
Raptor gene as a putative target of microRNA-210. (A) Genes putatively modulated in gene expression analysis by microRNA-210 in human erythroid induced cells. Representation of the intersections of microRNA-210 targets whose expression is down-regulated in ErPCs from Th17 (high HbF) *vs*. Th1 (low HbF) and down-regulated in MTH-induced K562 cells, but resulting up regulated by anti-microRNA-210. Arrowed three putative microRNA-210 target genes. (B) Location of the two microRNA-210 two binding sites in the 3’-UTR of raptor mRNA. (C) Structure of microRNA-210 and the predicted alignments with the 3’-UTR sequence of raptor mRNA and with three validated target mRNAs (PLK1, ROD1, E2F3).

A further comparative analysis was performed using RNA from erythroid progenitor cells isolated from Th1 (low HbF) and Th17 (high HbF) β-thalassemia patients (Fig 5A) [26]. Among the genes which were found to be expressed at low levels when Th17 was compared to Th1 (using as threshold a 4.00 fold change value), 227 were found to be possible target genes of microRNA-210. Targets of microRNA-210 were identified by available databases (Union of DIANA-microT; MiRanda microma.org; MiRanda miRBase; PicTar 4-way; PicTar 5-way; TargetScanS algorithms). Since our first interest was to identify sequences modulated by microRNA-210, we compared this list with a list of transcripts which were found to be both down-regulated in MTH-induced K562 cells (excluding the transcripts modulated in MTH-induced K562 cells plus anti-microRNA-588) and up regulated in MTH-induced K562 cells treated with anti-microRNA-210.

The comparison of these two datasets allowed the identification of three common genes, KIAA1303 (a sequence corresponding to raptor mRNA), FANK1 and CYB5R2 (Fig 5A). These genes were considered as putative targets of microRNA-210 in erythroid cells in association with increase of HbF production.

### Raptor, FANK1 and CYB5R2 mRNAs contain microRNA-210 binding sites

All the three genes, as expected, contain binding sites for microRNA-210, as found employing miRGator, as well as inspection of the previously published paper by Fasanaro *et al*. [41], describing an integrated approach to identify microRNA-210 target mRNA sequences. As an example, Fig 5B shows the raptor 3’-UTR location of microRNA-210 binding sites (evaluated using http://www.microrna.org/) together with sites for other microRNAs identified in Fig 2C and possibly associated with HbF expression. It should be underlined that two putative sites for microRNA-210 are present with the 3’-UTR of the human raptor mRNA (Fig 5B). The extent of complementarity between these sequences with mature microRNA-210 is 36.4% for microRNA-210-640 site and 72.7% for microRNA-210-1654 site (evaluated using TargetScanS 4.2, http://www.targetscan.org/). Accordingly, we can speculate that among the two microRNA-210 sites the most critical for raptor expression is microRNA-210-1654. The % of complementarity between microRNA-210 and raptor-1654 site is higher with respect to that found when FANK1 and CYB5R2 were considered (data not shown). Most importantly, this homology is similar or even higher than the homology between microRNA-210 and other validated target mRNAs (Fig 5C), such as PLK1 (polo-like kinase 1), ROD1 (Regulator of Differentiation 1) and E2F3 (E2F transcription factor 3) [42–44]. These analyses allowed us to concentrate our attention on raptor.

In addition, we were interested in raptor because the protein raptor is an important companion of mTOR, participating to the mTOR-C1 complex [39, 42–46]. This is relevant with respect to K562 cells erythroid induction, since we have previously published that rapamycin (RAPA, a drug specifically targeting mTOR through FK506 binding protein 1A) is a potent inducer of erythroid differentiation both in K562 cells and in ErPCs from β-thalassemia patients [34, 35–47]. Accordingly, we speculated that inhibition of raptor might also be a mechanism associated with promotion of differentiation of K562 cells and HbF production of ErPC cells (in this case induced by MTH). In our mind, a possible common basis, i.e. drug inhibition of the mTOR pathway (direct in the case of RAPA, raptor-mediated in the case of MTH) of activity of two inducers with complete different chemical structure was intriguing. This hypothesis prompted us to verify whether raptor mRNA is present in erythroid cells and whether its expression is inhibited following MTH treatment.

### Expression of microRNA-210 and raptor gene in K562 cells and erythroid precursors

**Fig 6:**
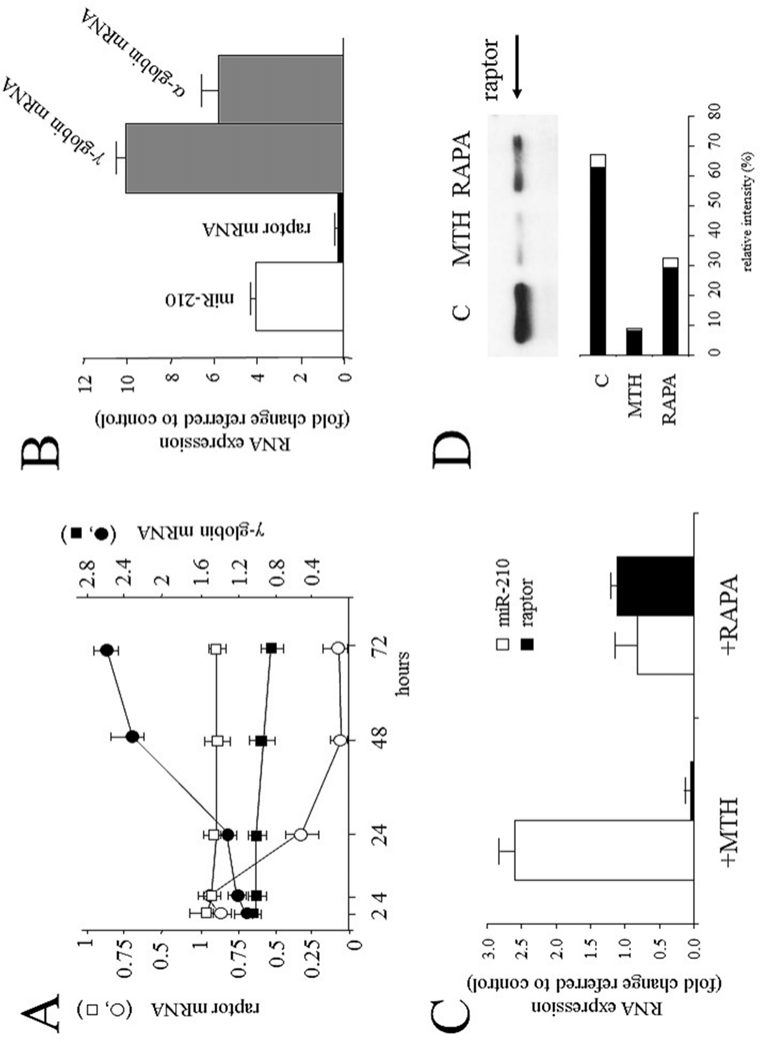
Quantification of microRNA-210, raptor, α- and γ-globin transcripts in MTH-induced K562 cells. (A) K562 cells were seeded at 30000/ml following treatment with 30 nM MTH and recovered at the indicated times. The raptor mRNA content is depicted as white symbols and γ-globin mRNA as black symbols. Untreated cells are depicted as squares and MTH-treated cells as circles. Results represent the fold changes with respect to untreated K562 cells at time = 0. (B) qPCR analysis of microRNA-210 (white histogram), raptor mRNA (black histogram), α- and γ-globin transcripts (grey histograms) in MTH-induced K562 cells after five days of erythroid differentiation (mean±S.D. from three different experiments). Results represent fold changes with respect untreated K562 cells. (C) K562 cells were treated with 30 nM MTH and 1 μM RAPA and analyzed after five days of induction. MicroRNA-210 and raptor mRNA content was calculated with respect to untreated cells using U6 snRNA or 18S as reference genes respectively (mean±S.D. from three different experiments). (D) Western blotting of the cellular extracts obtained from untreated K562 cells or K562 cells treated using 30 nM MTH and 1 μM RAPA for five days. Western Blotting was performed using antibodies against raptor protein (arrowed); in the lower part of panel D, the graph obtained by densitometric analysis of the autoradiograms is shown. Normalized intensity values are expressed as a percentage and mean (black) and standard deviation (white) from three independent experiments are reported. Ponceau S staining of the Western blots was used as the loading control.

Fig 6A demonstrates that raptor mRNA content decreases after 24 hours of MTH-treatment and this decrease is followed by an increase of γ-globin mRNA starting from low expression of this gene in uninduced K562 cells, strongly suggesting that the decrease of raptor gene expression is an early marker of MTH-mediated erythroid induction. Fig 6B shows a summary of three independent experiments performed in MTH-treated K562 cells after five days of MTH treatment, demonstrating that the increase of microRNA-210 was reproducibly associated with a decrease of raptor mRNA, and a parallel increase of α- and γ-globin mRNA accumulation. The expression of β-globin was not considered, since K562 cells are known to express the β-globin gene at undetectable or very low levels [34]. The embryonic ε-globin and ζ-globin mRNA are also induced in K562 cells, but the data were not included, since these genes are not activated in EPO-treated ErPCs and therefore are not useful for K562 cells-ErPC comparisons. The expression of other putative microRNA-210 target genes, such as FANK1 and CYB5R2, resulted unchanged or undetectable (data not shown). A further set of experiments was performed in order to compare MTH-mediated effects to those of RAPA after five days of treatment. No major effects of RAPA, well in agreement with its peculiar mechanism of action, were found on microRNA-210 and raptor mRNA content (Fig 6C); in fact, RAPA binds directly to mTORC1 by associating with its intracellular receptor FKBP12, without causing a reduction of raptor protein level [48]. The data described in panels A-C of Fig 6 have been confirmed by Western blotting analysis showing a dramatic decrease of raptor protein in MTH-treated cells. The decrease of the raptor protein is much less evident in RAPA-treated K562 cells (Fig 6D).

**Fig 7:**
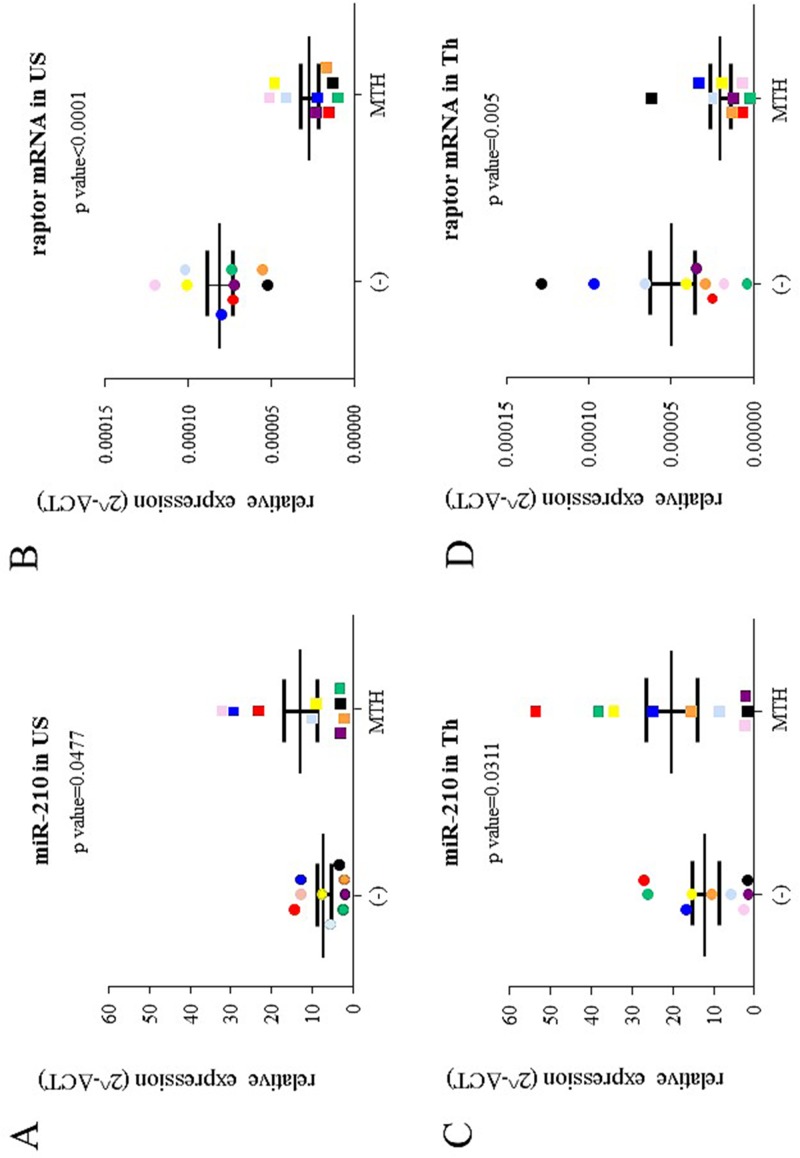
Expression of microRNA-210 and raptor mRNA in erythroid precursor cells. Quantification by RT-qPCR of microRNA-210 (A, C) and raptor mRNA (B, D) in 9 healthy donors (US) (A, B) and 9 β-thalassemia patients (Th) (C, D). The untreated ErPC samples are depicted as circles and the treated (30 nM MTH) as squares; samples from a same subject are identified by the same color. Means and standard deviations are reported. RT-qPCR analysis was performed after four days of erythroid differentiation. MicroRNA-let-7c (equally expressed in these samples) and 18S were used as internal reference genes to normalize RNA input.

In a second set of analyses, we have treated with MTH ErPCs from 9 healthy donors and 9 β-thalassemia patients. In this case we found, as expected, quite variable results. However, in most of the analyzed ErPC populations analyzed MTH induced, as expected, a significant increase of microRNA-210 (Fig 7, 7A and 7C). As found in MTH-treated K562 cells, raptor mRNA content sharply decreases following MTH treatment in most of the ErPCs samples following MTH treatment (Fig 7, 7B and 7D). In addition, in agreement with the data shown in the microarray and RT-qPCR analyses depicted in Fig 2, we would underline that the majority of β-thalassemia ErPCs express higher starting microRNA-210 levels than healthy ErPCs exhibiting at the same time lower raptor mRNA content. Biochemical features of the β-thalassemic ErPCs, including MTH-induced globin gene expression and genotype are reported in Table 2.

**Table 2:** Phenotype and genotype of the ErPCs from thalassemia patients employed in microRNA-210 and raptor analyses.

Patient number	α-globin mRNA (*)	β-globin mRNA (*)	γ-globin mRNA (*)	genotype
1	1.26	0.74	10.70	β^0^-39/β^+^-IVSI-110
2	1.27	1.14	5.13	β^0^-IVSI-1/β^+^-IVSI-6
3	0.72	2.05	1.80	β^0^-39/β^0^–39
4	1.09	0.95	2.14	β^0^-39/β^0^–39
5	1.11	0.96	3.05	β^+^-IVSI-6/β^+^-IVSI-110
6	1.04	1.13	3.39	β^0^-39/β^+^-IVSI-110
7	1.71	0.97	3.80	β^0^-39/β^0^–39
8	0.29	0.17	2.39	β^0^-39/β^0^-IVSI-6
9	1.13	2.35	4.29	β^0^-6(-A)/β^+^-IVSI-110

(*) fold increase in MTH-treated Th-ErPCs (with respect to untreated control cells)

### The down-regulated expression of raptor gene during MTH-induced erythroid differentiation of K562 cells is modulated by anti-microRNA-210

**Fig 8:**
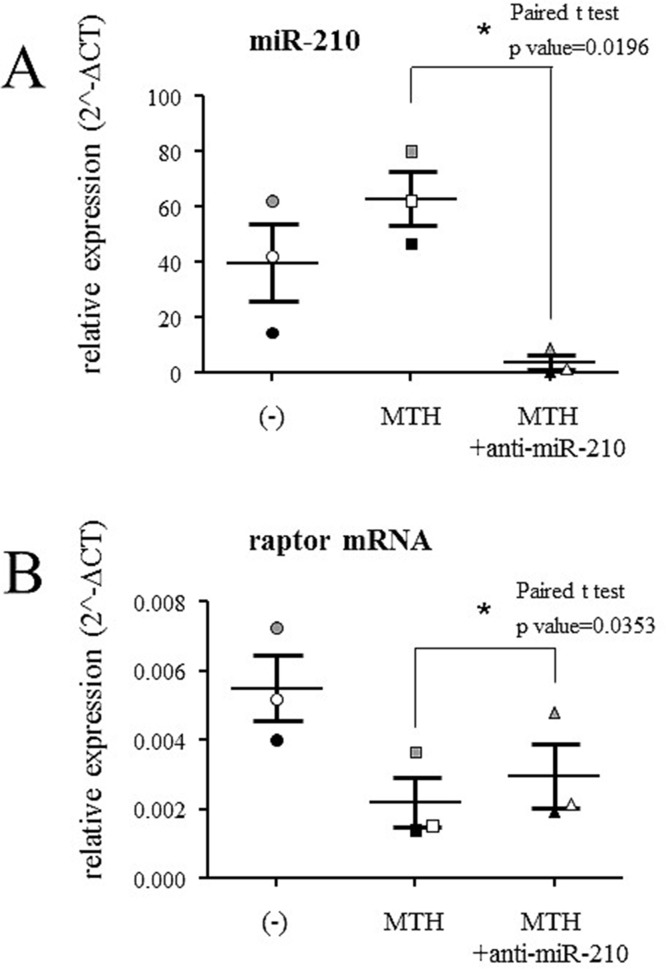
Expression of microRNA-210 and raptor mRNA in K562 cells. Quantification by RT-qPCR of microRNA-210 (A) and raptor mRNA (B) in K562 cell line was performed. Samples from uninduced cells are presented as circles, from 30 nM MTH-treated cells as squares and from cells treated with MTH + anti-microRNA-210 as triangles. RT-qPCR analysis was performed after five days of erythroid differentiation. The three different experiments performed are indicated in black, grey and white (* = p<0.05). Mean and standard deviation are reported. MicroRNA-let-7c (equally expressed in the samples) and 18S were used as internal reference genes to normalize RNA input.

Three independent experiments were performed treating K562 cells with MTH and with MTH plus anti-microRNA-210. After five days of treatment, RNA was extracted and the levels of microRNA-210 and raptor mRNA determined. The data obtained are shown in Fig 8A and confirm that microRNA-210 is up-regulated following MTH treatment. In addition, microRNA-210 is suppressed when anti-microRNA-210 was transfected to the cells. In parallel, while a strong MTH-mediated down-regulation of raptor mRNA was found, as expected, raptor mRNA increased when anti-microRNA-210 was added to MTH-treated cells (Fig 8B, p<0.05). This finding supports the hypothesis of an involvement of microRNA-210 in determining the raptor mRNA content.

### Effects of premicroRNA-210 on pmirGLO luciferase vectors incorporating the raptor microRNA-210 binding sites

**Fig 9:**
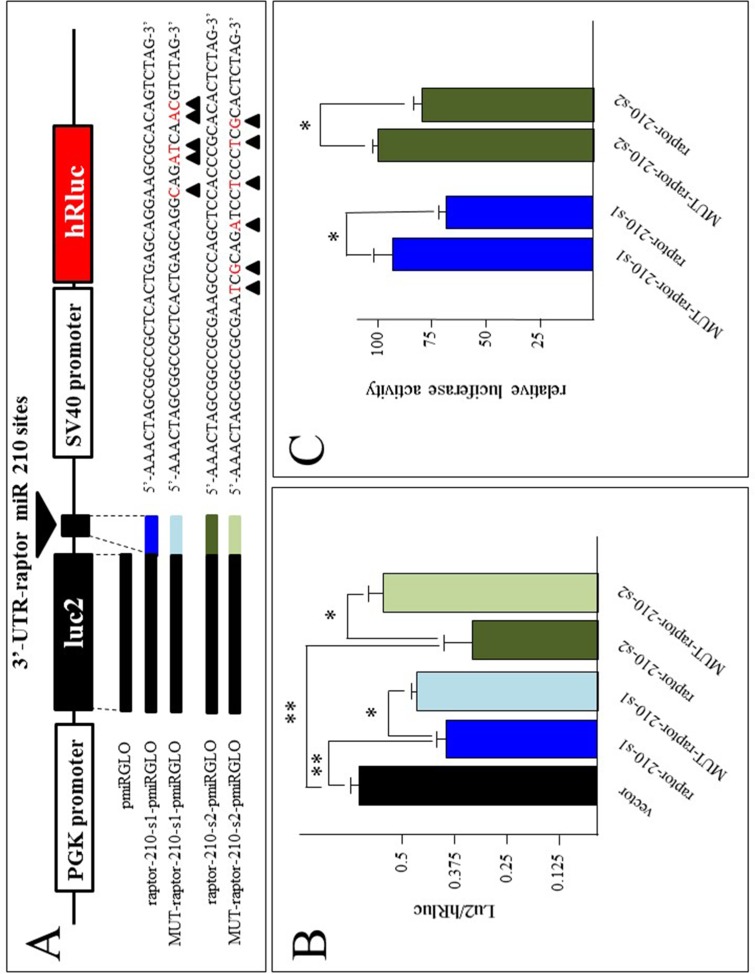
The raptor microRNA-210 sites modulate luciferase activity in a reporter gene construct. (A) Scheme of the pmirGLO vector, location and sequences of the cloned normal and mutated microRNA-210 sites in the raptor 3’-UTR. The arrowed nucleotides indicate the base substitution introduced in raptor target microRNA-210 mutated sites 1 and 2. (B) Luc2/hRluc ratios obtained in extracts of K562 cells treated with the pmirGLO vector or with the raptor-210-site1-pmiRGLO, MUT-raptor-210-site1-pmiRGLO, raptor-210-site2-pmiRGLO, MUT-raptor-210-site2-pmiRGLO vectors. (C) Relative luciferase activity in extracts treated with the raptor-210-site1-pmiRGLO, MUT-raptor-210-site1-pmiRGLO, raptor-210-site2-pmiRGLO, MUT-raptor-210-site2-pmiRGLO vectors in the presence of premicroRNA-210. The data represent the luciferase activity relative to K562 cells treated with MUT-raptor-210-site2-pmiRGLO vector.

These experiments were performed to verify whether raptor 3’-UTR mRNA sequences carrying the microRNA-210 binding sites might be a target site of microRNA-210. To this aim, we generated 4 constructs using the pmirGLO vector, carrying the firelyfly luciferase gene (luc2) under the control of the PGK promoter and the internal reference renilla luciferase (hRluc) under the control of the SV40 promoter. In this vector we cloned the two microRNA-210 raptor sequences (Fig 9A) and two mutated sequences carrying nucleotide substitution in evolutionary conserved positions. The structure of the pmirGLO vector, the cloning site, the sequences of the raptor 3’-UTR regions and the generated clones are reported in panel A of Fig 9. These clones were transfected to K562 cells in 4 independent experiments (all done in quadruplicate) and the luc2/hRluc ratio determined. The results obtained are shown in Fig 9B and clearly indicate that the luc2 expression driven by the raptor-210-s1-pmirGLO and raptor-210-s2-pmirGLO vectors is significantly lower than that driven by the reference pmirGLO vector (p<0.01). Remarkably, this effect is less evident when the mutated clones were employed (p<0.05). These results are compatible with a direct effect of the endogenous microRNA-210 on the microRNA-210 binding sites of the raptor 3’-UTR sequence. In order to further verify this hypothesis we forced microRNA-210 expression in K562 cells by transfection of premicroRNA-210 (Fig 9C), and co-transfection was performed with the vectors reported in Fig 9A. In these experiments the results were reported as relative luciferase activity using the data obtained with the mutated MUT-raptor-210-s2 as reference. The results clearly indicate that transfection with the premicroRNA-210 leads to a reduction of luc2 activity driven by both raptor-210-s1-pmirGLO and raptor-210-s2-pmirGLO vectors (Fig 9C). Altogether, these data support the concept that the microRNA-210 binding sites present within the 3’-UTR sequence of the raptor mRNA are direct targets of microRNA-210.

### Alteration of the mTOR pathway is associated with erythroid differentiation induced by MTH: comparison with the effects of the mTOR inhibitor rapamycin

**Fig 10:**
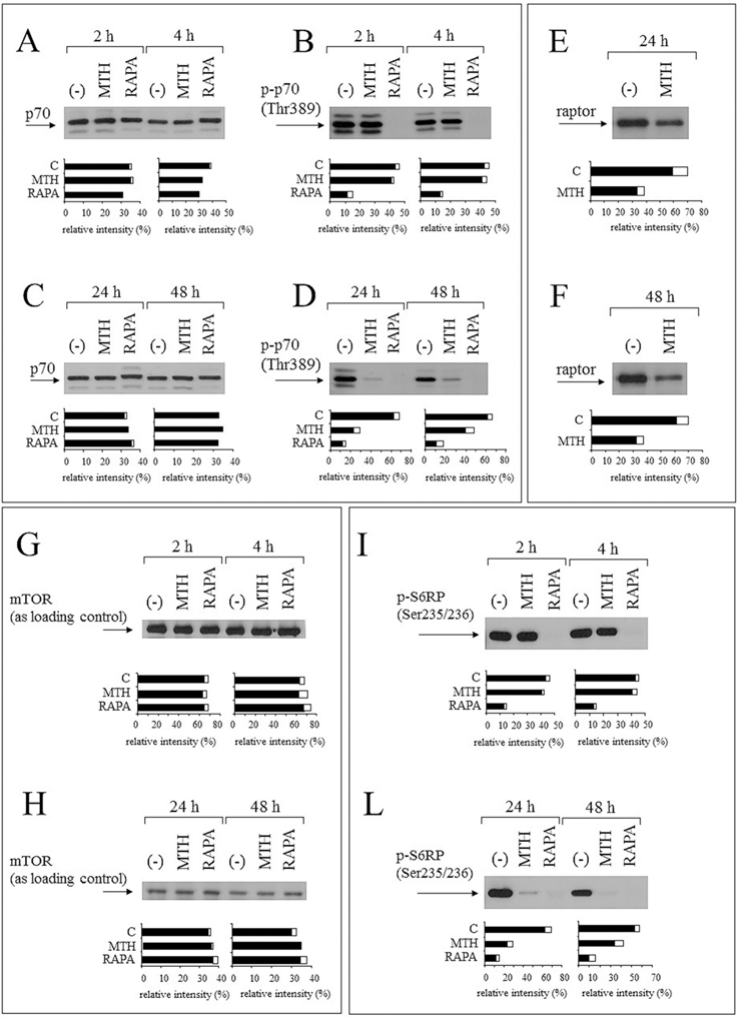
Comparison of the effects of MTH to RAPA on p70S6Kinase phosphorylation. (A-D) K562 cells were treated as indicated with 30 nM MTH and 1 μM RAPA and analyzed after 2, 4, 24, 48 hours by Western blotting using monoclonal antibodies against p70S6Kinase (A, C) and against the phosphorylation site of p-p70S6K Thr389 (B, D); (-) = untreated cells. (E, F) Determination of raptor protein production using specific monoclonal antibodies in untreated K562 cells and in K562 cells treated with MTH for 24 and 48 hours (when the modification of p70S6Kinase phosphorylation occurs). Western blotting was performed using antibodies against raptor protein (arrowed). G, H) Western blotting showing expression of mTOR, used as loading control in addition to Ponceau S staining. (I, L) The densitometric analysis of K562 cells were treated as indicated with 30 nM MTH and 1 μM RAPA and analyzed after 2, 4, 24, 48 hours by Western blotting using monoclonal antibodies against the phosphorylation site of p-S6RP Ser235/236. The quantitative densitometric analysis of all the autoradiograms is shown in the lower part of the panels. Normalized intensity values are expressed as the mean (black) and standard deviation (white) from three independent experiments.

In order to evaluate the possible targeting of the mTOR pathway by both MTH (through raptor) and RAPA (through mTOR binding by FKBP12), we compared the effects of these two inducers on the phosphorylation of p70S6Kinase (p70S6K), which specifically phosphorylates the ribosomal protein S6, strongly involved in sustaining protein synthesis and cell growth [49, 50]. To this aim, K562 cells were treated with MTH or RAPA for 2, 4, 24 and 48 hours, proteins isolated and p-p70S6K protein phosphorylated at Thr389 quantified by Western blotting (Fig 10, 10A–10D). The obtained results clearly indicate that RAPA-mediated inhibition of p70S6K phosphorylation is rapid and occurs within the first 2 hours (compare panel B with panel A), in agreement with the concept that the formation of mTOR-RAPA-FKBP12 complex leads to mTOR inhibition [51, 52]. On the contrary, MTH was not able to inhibit p70S6K phosphorylation after 2 and 4 hours; however, lower content of p-p70S6K is clearly evident after 24–48 hours (compare panel D with panel C), fully in agreement with the hypothesis that the inhibition of p70S6K phosphorylation is associated to raptor decrease in MTH-treated cells (Figs 6D, 10E and 10F). In these experiments mTOR was used as internal control (Fig 10G and 10H), because its expression was not altered by treatments.

Notably, mTOR-C1 complex is involved in the phosphorylation of the mTOR substrates p70S6K [53–55] and, by acting on p70S6K, it facilitates ribosome biogenesis and translation elongation [56, 57]. In addition, p70S6K1 can phosphorylate translational regulators such as eIF4B (eukaryotic translation initiation factor 4B) and PDCD4 (programmed cell death 4) to enhance the translational efficiency of mRNAs with highly structured 5′-UTRs [58, 59]. Similarly, raptor siRNA significantly decreases p70S6K1 phosphorylation [48, 60, 61]. Therefore, the phosphorylation status of p70S6K target ribosomal protein S6 (S6RP) was examined, since its phosphorylation sites could be altered by FKBP12-RAPA-mTOR or mTOR-raptor interactions [48, 62].

The comparison of the S6RP phosphorylation both in MTH- and RAPA-treated K562 cells is reported in Fig 10I and 10L, where a decrease of S6RP phosphorylation at Ser235 and Ser236 sites is evident. In addition to Ponceau S staining (reported as supplementary material), we use mTOR as loading control (Fig 10G and 10H). As far as timing, the results obtained by Western blotting analysis demonstrate that p-S6RP content decreases rapidly in RAPA-treated cells (2 hours treatment, see Fig 10I), and with a much slower kinetic (within 24–48 hours, see Fig 10L) in MTH-treated cells.

### Up-regulation of microRNA-210 and down-regulation of raptor mRNA during MTH erythroid induction of ErPCs from β-thalassemia patients

**Fig 11:**
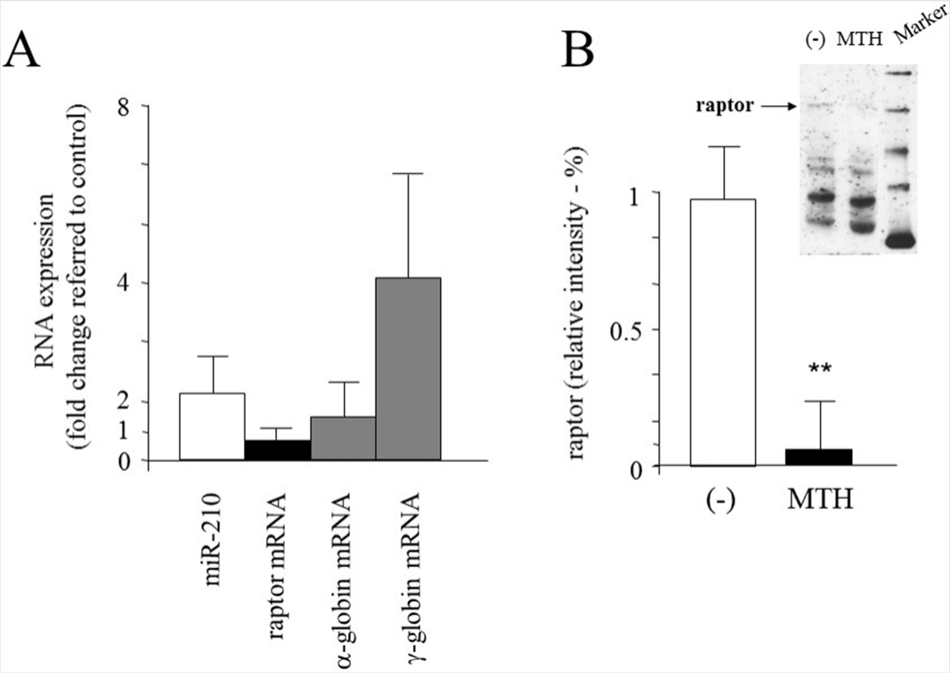
Effects of MTH treatment on ErPCs. (A) Modulation of microRNA-210, raptor, α- and γ-globin mRNAs in ErPCs from β-thalassemia patients by MTH. ErPCs obtained from 9 patients were grown in the two-phase liquid culture system. The effects were investigated in cells treated with 30 nM MTH for four days. The mRNA content was assayed by RT-qPCR using microRNA-let-7c (equally expressed in the samples) and 18S as reference gene sequences. The data represent mean±S.D. from 9 different experiments and are expressed as fold change with respect to untreated ErPCs (p<0.01, using One-way ANOVA, Kruskal-Wallis test). (B) Western blotting analysis of raptor protein in ErPCs from β-thalassemia patients, either untreated (-) or treated for four days with 30 nM MTH (p<0.05). The data represent the mean±S.D. of densitometric analysis from three independent experiments. In the upper insert, a representative example of Western blotting analysis.

In order to determine whether MTH affects globin gene expression together with microRNA-210 and raptor alteration in other erythroid cell model systems, we have considered ErPCs from peripheral blood of the β-thalassemia patients reported in Fig 7 and Table 2. Fig 11A shows the RT-qPCR analysis demonstrating an increase of microRNA-210 associated to reduction of raptor mRNA in MTH-treated ErPCs with respect to uninduced ErPCs employed as reference control, in agreement with the results obtained in MTH-induced K562 cells (see Fig 8). As expected, and elsewhere published [25], selective increase of γ-globin mRNA was also detected without major changes on the expression of α-globin genes. The data on the MTH-mediated effects on raptor mRNA are fully in agreement with the Western blotting analysis reported in Fig 11B, where a representative experiment is depicted in the insert (upper right side of Fig 11B) together with the data from three independent experiments performed on MTH-induced and uninduced ErPC lysates, using as probe a monoclonal antibody against human raptor. As clearly evident, the content of raptor protein is much lower in MTH-induced ErPCs.

### Anti-microRNA-210 reverses MTH-mediated inhibition of raptor mRNA content

**Fig 12:**
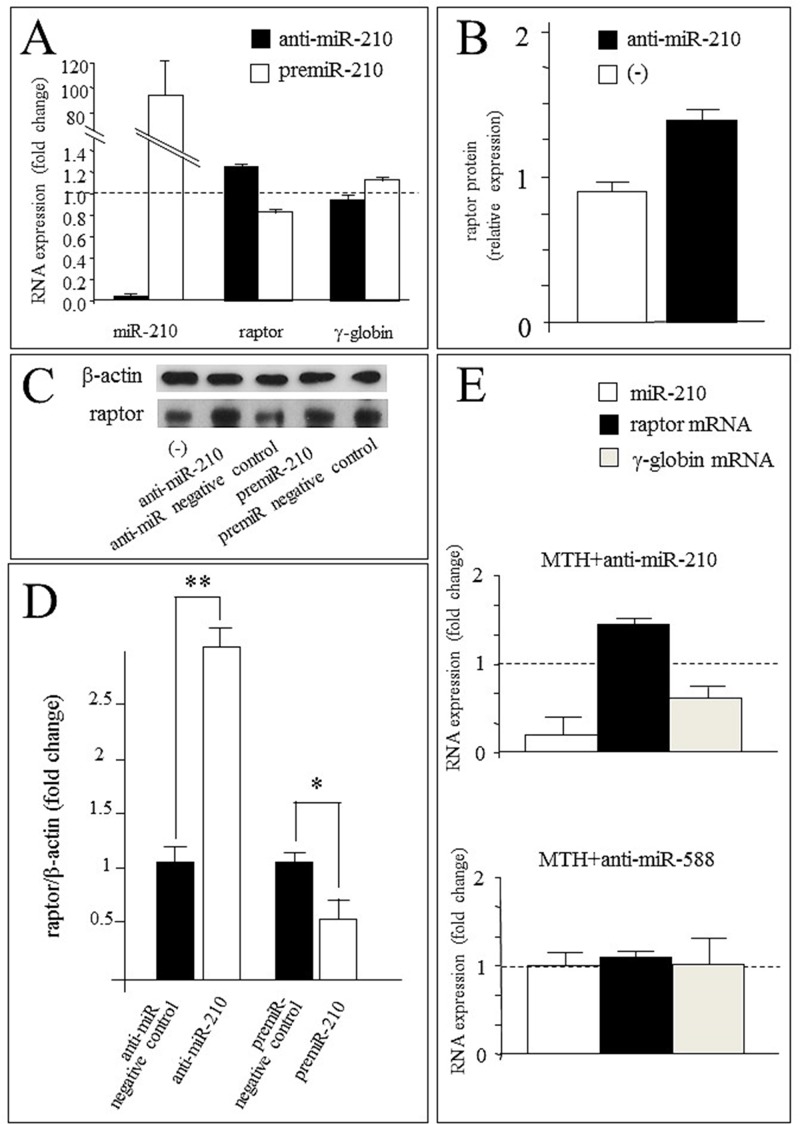
Effects of anti-microRNA-210 and premicroRNA-210 on K562 cells. (A) Effects of anti-microRNA-210 and premicroRNA-210 administration at the concentration of 200 nM with the siPort NeoFX transfection reagent (Ambion). The cells were seeded at 30000/ml and RNA extraction was carried out after five days of treatment. RT-qPCR analysis of microRNA-210, raptor and γ-globin transcripts was performed using untreated cells as reference and microRNA-let-7c and 18S as internal control gene sequences. The data represent the mean±S.D. from three independent experiments. (B) Effects of anti-microRNA-210 administration on raptor protein content in uninduced K562 cells and analyzed by Western blotting. The data represent the mean±S.D. from three independent experiments. (C) Effects of anti-microRNA-210 and premicroRNA-210 administration on raptor protein content in K562 cells analyzed by Western blotting. Beta-actin was used as the internal control. (D) Densitometric Western blotting analysis (as that shown in panel C) reporting the ratio raptor/β-actin. The data represent the mean±S.D. from three independent experiments (* = p<0.05; ** = p<0.01). (E) Analysis of microRNA-210, raptor and γ-globin transcripts in K562 cells induced to erythroid differentiation by MTH and treated with anti-microRNA-210 or with anti-microRNA-588 (taken as negative control). RT-qPCR data are presented as fold changes in respect to MTH-treated cells, using microRNA-let-7c and 18S as reference gene sequences. The data represent the mean±S.D. from three independent experiments.

The use of anti-microRNA targeting microRNAs and siRNAs recognizing mRNAs might help to understand the involvement of microRNAs and mRNAs in biological functions as published in several reports [63–65]. In this context, we first analyzed the effects of anti-microRNA-210 and premicroRNA-210 on the expression of microRNA-210, raptor and globin transcripts in uninduced K562 cells. Anti-microRNA-210 and premicroRNA-210 were administered daily at the concentration of 200 nM with the siPort NeoFX transfection reagent (Ambion, Applied Biosystems, Foster City, CA, USA). The results reported in Fig 12A demonstrate that anti-microRNA-210 strongly reduces microRNA-210 content, in association with an increase of raptor mRNA. As expected, the effects of premicroRNA-210 administration were found to be opposite (a sharp increase of microRNA-210 was associated with a decrease of raptor mRNA in uninduced K562 cells). The results on raptor mRNA were in agreement with the Western blotting analysis reported in Fig 12B, showing a higher content of raptor protein following treatment of K562 cells with anti-microRNA-210 (1.41±0.12, fold increase with respect to the untreated controls). These changes of target mRNA content following modulation of microRNAs are in line with the extent of changes found by other research groups in other experimental systems and probably associated to control of a single target mRNAs by several microRNAs [66–68].

In order to conclusively analyze the effects of anti-miR-210 and premiR-210 on raptor gene expression, Western blotting experiments were conducted and the results shown in Fig 12 (12C and 12D). K562 cells were transfected with 400 nM anti-microRNA-210 and premicroRNA-210, cytoplasmic protein extracts were prepared after 2 days of cell culture and Western blotting analysis performed, using β-actin as reference internal control. The results obtained confirmed that anti-miR-210 treatment leads to a sharp increase of raptor protein expression; conversely, raptor expression decreases following premiR-210 treatment.

In a second set of experiments, the effects of anti-microRNA-210 on expression of microRNA-210, raptor and globin transcripts in MTH-induced K562 cells were analyzed. In this case, an increase of raptor mRNA was found associated to a sharp decrease of microRNA-210 content (Fig 12E). Interestingly, in MTH-treated cells a reduction of γ-globin mRNAs is observed together with the microRNA-210 decrease and raptor mRNA increase (Fig 12E). These data are fully in agreement with the hypothesis of an involvement of microRNA-210 with γ-globin gene expression. When the effects of anti-microRNA-210 were compared with those of anti-microRNA against microRNA-588 (used as control), we observed that this anti-microRNA molecule exhibited no effects on microRNA-210, raptor mRNA and γ-globin mRNA content (Fig 12E), allowing us to conclude that the effects of anti-microRNA-210 were specific.

### Sequence-specific silencing of raptor gene enhances MTH-mediated effects on γ-globin gene expression

**Fig 13:**
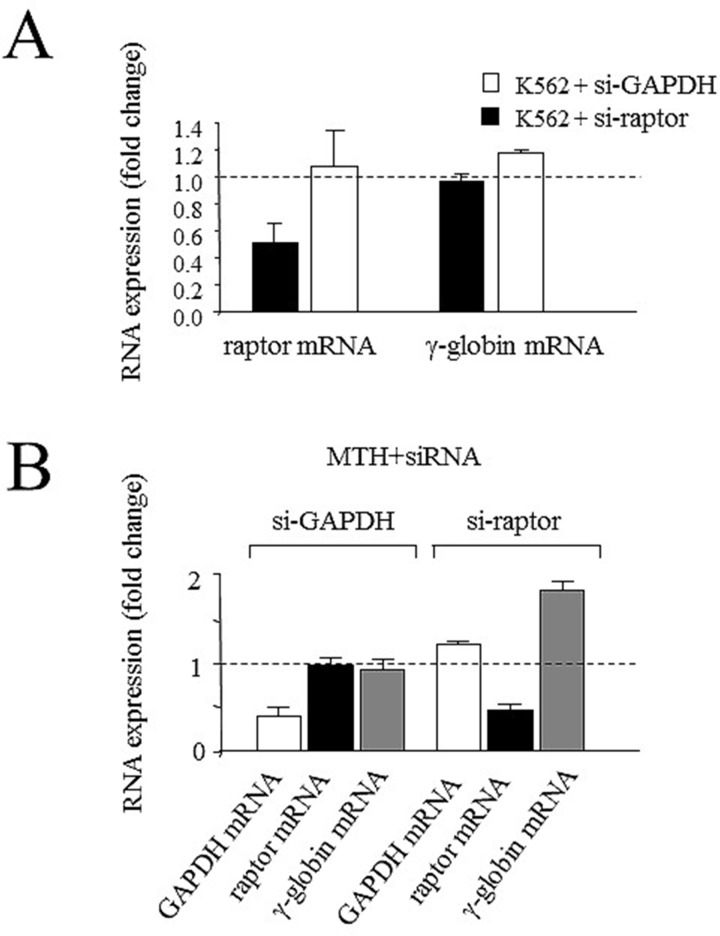
Effects of siRNA against raptor mRNA on K562 cells γ-globin gene expression. (A) Treatment of uninduced K562 cells with siRNA against raptor mRNA (black boxes) and siRNA against GAPDH mRNA (white boxes). (B) Effects of siRNA against raptor mRNA and siRNA against GAPDH mRNA on K562 cells induced to erythroid differentiation using a suboptimal concentration of MTH (15 nM). K562 cells were seeded at 10000 cell/100 μl following the Ambion transfection protocol using siPort NeoFX transfection reagent and 200 nM Silencer GAPDH siRNA and pre-designed siRNAs targeting raptor mRNA. Cells-to-cDNA II Kit was used to produce cDNA and RT-qPCR were conduced as reported in the methods section. The histograms represent the effects of si-GAPDH and si-raptor mRNA on GAPDH (white boxes), raptor (black boxes) and γ-globin (grey boxes) transcripts. The data represent the mean±S.D. from three independent experiments.

In order to investigate the possible role of down-regulation of raptor mRNA on γ-globin mRNA expression during MTH-induced erythroid differentiation, uninduced K562 cells and K562 cells treated with a suboptimal concentration of MTH (15 nM) were transfected with siPort NeoFX transfection reagent using 200 nM Silencer GAPDH siRNA and pre-designed siRNA targeting raptor mRNA (catalog number ID: s33214, Applied Biosystems, Foster City, CA, USA) as described in experimental procedure and siRNA effects evaluated (Fig 13). The low suboptimal MTH concentration has been chosen to avoid the induction of high proportion of benzidine-positive cells, thereby allowing identification of possible synergistic effects in association with specific treatments. SiRNA against raptor mRNA in uninduced K562 cells leads to a specific reduction of raptor mRNA levels in uninduced K562 cells, in the absence of any modulation of γ-globin mRNA content (Fig 13A). On the contrary, si-raptor transfection to K562 cells treated with the suboptimal concentration of MTH induces a decrease of raptor mRNA associated with a sharp increase of γ-globin gene expression (Paired t-test, two tailed, p = 0.0274). When si-GAPDH was employed, inhibition of GAPDH mRNA was observed in the absence, as expected, of major effects on raptor mRNA and γ-globin mRNA both in untreated (Fig 13A) and MTH-treated (Fig 13B) K562 cells. The findings reported in Fig 13 allow us to conclude that the effects of siRNA against raptor are specific and lead to increase of γ-globin mRNA expression only in K562 cells induced to erythroid differentiation using suboptimal concentration of the inducer agent.

## Discussion

The involvement of microRNAs in erythroid hematopoiesis has been recently proposed by several studies performed on very different experimental systems, including erythroid-induced UT-7 [23] and K562 cell lines [20, 22, 69, 70], human umbilical cord blood (UCB) CD34^+^ cells [11, 12], CD34^+^ hematopoietic stem-progenitor cells (HSPCs) from human bone marrow [13, 16], human ErPCs from peripheral blood [22], and zebrafish primitive erythropoiesis [71].

We and others have reported that microRNA-210 is involved in erythroid differentiation and, possibly, in γ-globin gene up-regulation [23, 27, 72]. Kosaka *et al*. reported the identification of erythropoietin-induced microRNAs in erythropoietic cells during erythroid differentiation using the erythropoietin-dependent cell line UT-7 as a model system [23], but extending their analysis to primary erythroid cells, such as fetal liver erythroid cells. Their conclusion was that microRNA-210 is involved in erythroid differentiation and maturation. High levels of microRNA-210 were also found by other research groups [40, 41, 72–76].

In this study, we have firstly analyzed the expression of microRNA-210 in K562 cells induced to erythroid differentiation by the HbF inducer mithramycin (MTH), and found that microRNA-210 content increases following MTH-induction. In respect to the involvement on erythroid functions, the major conclusions of the present study are the following: (a) microRNA-210 expression is increased in ErPCs from a β-thalassemia patients expressing high levels of HbF (Fig 2A, 2D and 2F); (b) microRNA-210 expression increases following MTH-treatment of K562 cells and human ErPCs from β-thalassemia patients (Fig 2B and 2E, Figs 7 and 8); (c) this increase in microRNA-210 is associated with erythroid induction and elevated expression of γ-globin genes (Fig 2E and 2F, Figs 6B and 12A); (d) anti-microRNA-210 interferes with the MTH-induced changes of gene expression in MTH-treated K562 cells (Figs 3, 4 and 12E). These data strongly support the concept that microRNA-210 is involved in erythropoiesis and possibly in up regulation of the expression of γ-globin genes.

Relevant to our study, hypoxic conditions are associated with erythroid maturation and preferential activation of HbF. This has been reported by Narayan *et al*., who studied the effects of hypoxia on hemoglobin switching using hematopoietic progenitor cells from human fetal liver (F-LVR), cord blood (CB), and adult bone marrow (ABM). These authors found that HSCs from CB and F-LVR generated the same Hb profile under normoxia and hypoxia. HSCs from ABM had single-positive HbA and double-positive HbA and HbF cells at normoxia and almost entirely double-positive cells at hypoxia. They concluded that Hb switching is affected by the environment but not all HSCs are preprogrammed to respond [76].

In this respect it is interesting to note that microRNA-210 is among the microRNAs which are clearly up regulated in hypoxic conditions [39, 72] as recently published by Noman *et at*. [73]. The microRNA signature in hypoxia has been studied by Kulshreshtha *et al*. [37], Camps *et al*. [74] and Pulkkinen *et al*. [39]. Following these studies, microRNA-210 is a master regulator of gene expression in hypoxic conditions. This appears to be a general feature common to many cellular model systems [76, 77].

Taken together, our data suggest that MTH-induced erythroid differentiation, HbF induction and hypoxic conditions might display common molecular biology features, one of which is represented by microRNA-210 up-regulation. Therefore, in addition of the already reported mechanisms of action of MTH (which for instance is known to directly bind to the γ-globin gene promoter [24], MTH might exert its effects through microRNA-210 activation.

In respect to the identification of possible targets of microRNA-210, our study allowed us to identify raptor mRNA as (a) a sequence containing a 3’-UTR region with several microRNA-binding sites including two binding sites for microRNA-210 (Fig 5B); (b) expressed at low levels in ErPCs from a β-thalassemia patients producing high levels of HbF (Fig 5A); (c) down-regulated during MTH-induction of K562 cells and ErPCs from β-thalassemia patients (Figs 3A, 6B, 7B and 7D, Figs 8B and 11). Accordingly, the protein raptor decreases following MTH-mediated erythroid induction of K562 cells and ErPCs.

In order to obtain information about a possible functional role of raptor microRNA-210 binding sites, the 640-microRNA-210 and 1645-microRNA-210 binding sites (see Fig 5B and 5C), and the related mutated sequences (Fig 9) were cloned in the pmirGLO vector, carring the firelyfly luciferase gene (luc2) under the control of the PGK promoter and the internal control renilla luciferase (hRluc) under the control of the SV40 promoter. The data obtained suggest that the raptor microRNA-210 binding sites are direct targets of microR-210. Studies were also performed on K562 cells with the aim of quantifying raptor mRNA and raptor protein content following treatment with anti-microRNA-210 and premicroRNA-210. In the presence of anti-microRNA-210 the following effects in MTH-treated K562 cells were observed: (a) a decrease of microRNA-210 content (it should be underlined that the effects of anti-microRNA might be based on binding to mature microRNA-210, but also to primicroRNA or premicroRNA) (Figs 3B, 8A and 12); (b) an increase of raptor mRNA (Figs 5A, 8B and 12) and (c) a decrease of functions associated with differentiation processes (i.e. proportion of benzidine-positive cells, Hb, γ-globin mRNA accumulation) (Figs 3, 4 and 12E). When K562 cells were treated with premicroRNA-210 a decrease of raptor mRNA and raptor protein was detected. Altogether, these results strongly suggest raptor mRNA as a microRNA-210 target, despite the fact that we can not exclude that other molecular microRNA-210 targets might be involved in erythroid differentiation and γ-globin gene expression.

A final remark should be done on the implications of our results with respect to the relationship between mTOR pathway and erythroid differentiation. It should be underlined that rapamycin, a direct inhibitor of mTOR, exhibits high inducing effects on erythroid differentiation associated with a fast alteration of mTOR-regulated functions, including inhibition of phosphorylation of p70S6K, inhibition of phosphorylation of ribosomal protein S6 [54, 58, 62, 78–79]. Also, MTH induces the same effects possibly inhibiting raptor mRNA and protein with a slower kinetics, confirming that mTOR pathway is crucial for erythroid differentiation induced by MTH and RAPA. The effects of MTH on mTOR are conclusively supported by the findings that mTOR downstream biological functions are deeply altered by MTH, including phosphorylation of the mTOR substrates p70S6K and phosphorylation of the p70S6K target ribosomal protein S6 (S6RP) (Fig 10).

Despite the fact that silencing of raptor is not sufficient to induce up regulation of the expression of γ-globin genes, but needs the activation of other MTH-induced pathways (Fig 13), the data reported in the present study allow us to propose a link between microRNA-210, raptor, mTOR-C1, erythroid differentiation and preferential expression of the γ-globin genes in erythroid cells.

## Supporting Information

S1 Fig Treatment of K562 cells with 30 nM MTH and 1 μM RAPA after 2, 4, 24, 48 hours. An example of Western blotting using 10 μg of cytoplasmic extracts after Ponceau S Solution prestaining. The kDa of marker proteins are arrowed.
(TIF)

S1 Table MicroRNAs differentially expressed between thalassemic patients with high HbF vs. normal donors and thalassemic patients with low HbF. (DOC)

S2 Table MicroRNAs differentially expressed between K562+MTH *vs*. K562 control. (DOC)

S3 Table Significant pathways enriched in the list of 748 modulated mRNAs in K562 cells induced by MTH and up-regutaled by anti-miR-210. (DOC)
