# Supplementary figures and images for: Increase of microRNA-210, Decrease of Raptor Gene Expression and Alteration of Mammalian Target of Rapamycin Regulated Proteins following Mithramycin Treatment of Human Erythroid Cells
Source: PLoS One. 2015 Apr 7;10(4):e0121567. doi: 10.1371/journal.pone.0121567 (PMC4388523; doi:10.1371/journal.pone.0121567)

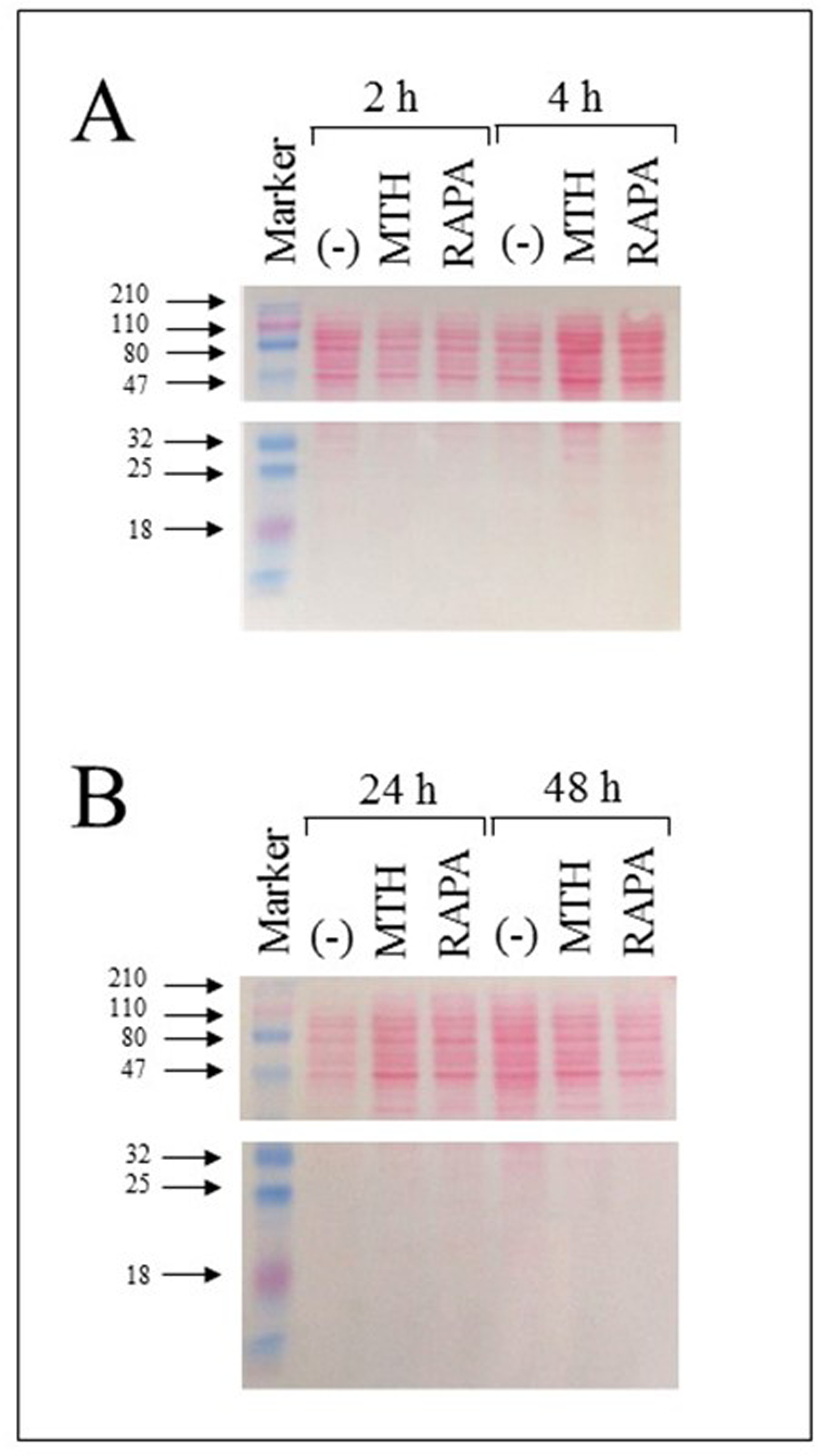

Supplement: S1 Fig — An example of Western blotting using 10 μg of cytoplasmic extracts after Ponceau S Solution prestaining. The kDa of marker proteins are arrowed. (TIF) [file pone.0121567.s001.tif]
