# Supplementary material for: Increase of microRNA-210, Decrease of Raptor Gene Expression and Alteration of Mammalian Target of Rapamycin Regulated Proteins following Mithramycin Treatment of Human Erythroid Cells
Source: PLoS One. 2015 Apr 7;10(4):e0121567. doi: 10.1371/journal.pone.0121567 (PMC4388523; doi:10.1371/journal.pone.0121567)
Supplement: S1 Table — (DOC) [file pone.0121567.s002.doc]

| **Systematic Name** | **regulation** | **p-value** | **FCAbsolute** | **Th high HbF (normalized)** | **US+Th low HbF (normalized)** |
| --- | --- | --- | --- | --- | --- |
| hsa-miR-877* | down | 0.00624935 | 4.8909674 | -0.07322968 | 2.2168899 |
| hsa-miR-630 | down | 0.04612583 | 2.4819553 | 5.5027986 | 6.8142753 |
| hsa-miR-124 | up | 0.03957665 | 5.1560497 | 6.2317257 | 0.54353124 |
| hsa-miR-574-5p | up | 0.00769811 | 4.5316586 | 3.2692907 | 1.0892513 |
| hsa-miR-148a | up | 0.01028958 | 4.2898436 | 6.732021 | 4.631096 |
| hsa-miR-15a | up | 0.00718574 | 3.2994204 | 7.2207627 | 5.498551 |
| hsa-miR-361-3p | up | 0.00594286 | 3.2579877 | 2.036053 | 0.3320717 |
| hsa-let-7i | up | 0.03090401 | 3.2304995 | 8.097230 | 6.4054728 |
| hsa-miR-155 | up | 0.01456532 | 3.1888871 | 3.2910607 | 1.6180077 |
| hsa-miR-29b | up | 0.00541529 | 3.1193159 | 4.9459558 | 3.3047261 |
| hsa-miR-27b | up | 0.04252342 | 3.1142218 | 4.3286057 | 2.6897337 |
| **hsa-miR-210** | **up** | **0.03657616** | **3.1101155** | **9.157292** | **7.5203247** |
| hsa-miR-142-5p | up | 0.02958235 | 2.8934026 | 6.931463 | 5.398695 |
| hsa-miR-30e* | up | 0.02031630 | 2.8229337 | 4.995385 | 3.49819 |
| hsa-miR-142-3p | up | 0.03471614 | 2.7838056 | 10.706464 | 9.229406 |
| hsa-miR-590-5p | up | 0.02305275 | 2.7165027 | 4.9179416 | 3.476191 |
| hsa-miR-26a | up | 0.00136061 | 2.6862226 | 7.4464607 | 6.0208817 |
| hsa-miR-503 | up | 0.02954496 | 2.6732492 | 1.3074993 | -0.11109505 |
| hsa-miR-421 | up | 0.03255004 | 2.5590575 | 1.6318034 | 0.2761907 |
| hsa-miR-454 | up | 0.03266178 | 2.4117875 | 3.1432056 | 1.8731028 |
| hsa-miR-28-5p | up | 0.02679212 | 2.3421261 | 2.7474353 | 1.5196166 |
| hsa-miR-30d | up | 0.01512367 | 2.2289581 | 8.424004 | 7.2676344 |
| hsa-miR-30a | up | 0.02191173 | 2.2140162 | 4.2572246 | 3.110559 |
| hsa-miR-22 | up | 0.01323448 | 2.1982615 | 5.868879 | 4.7325153 |
| hsa-miR-29c | up | 0.02327299 | 2.1890202 | 7.3344064 | 6.204121 |
| hsa-miR-342-3p | up | 0.00613448 | 2.1464646 | 7.3639464 | 6.261984 |
| hsa-miR-103 | up | 0.00455794 | 2.0908644 | 9.211688 | 8.147589 |
| hsa-miR-148b | up | 0.04455318 | 2.0836356 | 4.0300517 | 2.9709487 |
| hsa-miR-34a | up | 0.02894693 | 2.0637603 | 6.5237465 | 5.478471 |
| hsa-miR-769-5p | up | 0.01371183 | 2.0085466 | 3.007702 | 2.0015502 |
| hsa-miR-363 | up | 0.00939767 | 7.7478850 | 4.00501 | 1.0512078 |
| hsa-miR-222 | up | 0.01109592 | 5.2032420 | 3.6615580 | 1.282147 |
| hsa-miR-424 | up | 0.01501956 | 4.8916870 | 7.4997764 | 5.209444 |
| hsa-miR-197 | up | 0.00263326 | 3.6322960 | 3.9329803 | 2.0720985 |
| hsa-miR-30e | up | 0.01079358 | 3.3917080 | 7.759594 | 5.9975824 |
| hsa-miR-29a | up | 0.00599770 | 2.8470440 | 8.042888 | 6.533423 |
| hsa-miR-140-5p | up | 0.01787434 | 2.8322270 | 6.333861 | 4.8319235 |
| hsa-miR-301a | up | 0.01865955 | 2.8167420 | 7.285992 | 5.791965 |
| hsa-miR-146a | up | 0.00118507 | 2.6083180 | 3.3382082 | 1.9550884 |
| hsa-miR-7-1* | up | 0.03029410 | 2.4799020 | 1.4985746 | 0.1882915 |
| hsa-miR-29c* | up | 0.01966725 | 2.4246920 | 1.4641193 | 0.18631783 |
| hsa-miR-16 | up | 0.00322942 | 2.2778630 | 10.099518 | 8.911837 |
| hsa-miR-30b | up | 0.01635884 | 2.0850860 | 7.6387763 | 6.578669 |
