# Supplementary material for: Increase of microRNA-210, Decrease of Raptor Gene Expression and Alteration of Mammalian Target of Rapamycin Regulated Proteins following Mithramycin Treatment of Human Erythroid Cells
Source: PLoS One. 2015 Apr 7;10(4):e0121567. doi: 10.1371/journal.pone.0121567 (PMC4388523; doi:10.1371/journal.pone.0121567)
Supplement: S2 Table — (DOC) [file pone.0121567.s003.doc]

| **Systematic Name** | **regulation** | **FCAbsolute** | **MTH (normalized)** | **C (normalized)** |
| --- | --- | --- | --- | --- |
| hsa-miR-638 | down | 24.544716 | 1.394474 | 6.0118146 |
| hsa-miR-572 | down | 16.645763 | -2.1021416 | 1.9549413 |
| hsa-miR-671-5p | down | 15.918625 | -1.7835760 | 2.2090678 |
| hsa-miR-1225-5p | down | 14.096669 | 2.65328 | 6.4705625 |
| hsa-miR-99b* | down | 13.623967 | -2.1021416 | 1.6659334 |
| hsa-miR-1224-5p | down | 10.1068945 | -1.4199009 | 1.917367 |
| hsa-miR-663 | down | 9.926618 | -1.0222346 | 2.2890677 |
| hsa-miR-564 | down | 9.580272 | -1.5467474 | 1.7133191 |
| hsa-miR-150* | down | 9.247061 | -2.1021416 | 1.1068532 |
| hsa-miR-134 | down | 7.8935657 | 0.5117059 | 3.492383 |
| hsa-miR-887 | down | 6.696147 | -2.1021416 | 0.6411896 |
| hsa-miR-940 | down | 5.623029 | 3.1799314 | 5.671279 |
| hsa-miR-483-5p | down | 5.5630474 | -0.2963456 | 2.1795297 |
| hsa-miR-708 | down | 5.495414 | -2.1021416 | 0.3560865 |
| hsa-miR-1226* | down | 5.30084 | -2.1021416 | 0.3040793 |
| hsa-miR-874 | down | 5.1005125 | 0.9099426 | 3.2605848 |
| hsa-miR-371-5p | down | 4.800687 | 0.30894566 | 2.5721865 |
| hsa-miR-654-5p | down | 4.680658 | -2.1021416 | 0.124569654 |
| hsa-miR-498 | down | 4.4243336 | -2.1021416 | 0.04331851 |
| hsa-miR-135a* | down | 4.408382 | -2.1021416 | 0.038107634 |
| hsa-miR-575 | down | 4.251407 | 2.0756009 | 4.1635413 |
| hsa-miR-630 | down | 3.851084 | 0.77076983 | 2.7160344 |
| hsa-miR-601 | down | 3.7368479 | -1.2353365 | 0.6664853 |
| hsa-miR-602 | down | 3.0665584 | -1.524459 | 0.09216142 |
| hsa-miR-188-5p | down | 2.8261423 | 1.6146188 | 3.113453 |
| hsa-miR-923 | down | 2.5691752 | 8.582975 | 9.944281 |
| hsa-miR-7 | down | 2.4769838 | 2.5745208 | 3.8831053 |
| hsa-miR-494 | down | 1.9680538 | 2.2599423 | 3.236712 |
| hsa-miR-628-3p | down | 1.9665159 | -0.67898834 | 0.2966535 |
| hsa-miR-939 | down | 1.9285419 | 3.752694 | 4.7002044 |
| hsa-miR-144 | up | 10.367723 | 5.7708473 | 2.39682 |
| hsa-miR-144* | up | 7.0984755 | 3.1794116 | 0.35190248 |
| hsa-miR-451 | up | 6.183976 | 9.100191 | 6.4716563 |
| hsa-miR-589* | up | 4.7397575 | 0.0040290356 | -2.2407842 |
| hsa-miR-182* | up | 3.639922 | 0.9001558 | -0.9637517 |
| hsa-miR-125a-5p | up | 2.605771 | 3.531862 | 2.1501517 |
| hsa-miR-181c* | up | 2.516971 | 0.10773587 | -1.2239528 |
| hsa-miR-574-3p | up | 2.3403614 | 1.4044096 | 0.17767835 |
| hsa-miR-196a | up | 2.3386524 | 4.3247185 | 3.099041 |
| hsa-miR-183* | up | 2.3058264 | 2.1349938 | 0.9297099 |
| hsa-miR-96 | up | 2.2704933 | 7.148489 | 5.965483 |
| hsa-miR-27a | up | 2.2681637 | 7.772691 | 6.591166 |
| hsa-miR-32 | up | 2.2644608 | 3.4652913 | 2.2861238 |
| hsa-miR-33a | up | 2.255486 | 3.217057 | 2.0436187 |
| hsa-miR-10a* | up | 2.1859958 | 0.44192338 | -0.6863673 |
| hsa-miR-374a | up | 2.1646092 | 7.073819 | 5.9597125 |
| hsa-miR-29b-1* | up | 2.1225994 | 0.8512707 | -0.23456144 |
| hsa-miR-99b | up | 2.1147225 | 3.2954319 | 2.2149634 |
| hsa-miR-199b-5p | up | 2.079284 | 0.8127215 | -0.24336529 |
| hsa-miR-129-3p | up | 2.015221 | 0.10132599 | -0.9096122 |
| hsa-miR-421 | up | 2.0066116 | 2.891157 | 1.8863955 |
| hsa-miR-182 | up | 2.0010686 | 4.101083 | 3.1003122 |
| hsa-miR-26a | up | 1.9785914 | 5.876626 | 4.8921523 |
| hsa-miR-200c | up | 1.9678739 | 1.2934697 | 0.31683183 |
| hsa-miR-183 | up | 1.9151547 | 5.7206974 | 4.7832365 |
| hsa-miR-195 | up | 1.9068485 | 3.850455 | 2.9192648 |
| **hsa-miR-210** | **up** | **1.9024541** | **5.486191** | **4.558329** |
| hsa-miR-30e | up | 1.8699014 | 7.1518974 | 6.248935 |
| hsa-miR-193b | up | 1.8698632 | 4.0318804 | 3.1289477 |
| hsa-miR-33b | up | 1.8475913 | 0.6158035 | -0.26984215 |
| hsa-miR-30b | up | 1.8420855 | 6.312104 | 5.430764 |
| hsa-miR-628-5p | up | 1.8215482 | 1.3083625 | 0.44319725 |
| hsa-miR-542-3p | up | 1.8108281 | 0.50983334 | -0.3468163 |
| hsa-miR-193a-3p | up | 1.8052324 | 2.6970546 | 1.8448701 |
| hsa-miR-424 | up | 1.8010365 | 4.8473816 | 3.9985542 |
| hsa-miR-181c | up | 1.7937031 | 0.7490957 | -0.09384537 |
| hsa-miR-101 | up | 1.7806896 | 5.957344 | 5.124908 |
| hsa-miR-148a | up | 1.7801777 | 5.1886415 | 4.3566203 |
| hsa-miR-30e* | up | 1.7767025 | 4.11979 | 3.290588 |
| hsa-let-7b* | up | 1.7698132 | 0.17874622 | -0.64485085 |
| hsa-miR-532-3p | up | 1.7560827 | 1.4855113 | 0.67315054 |
