# Supplementary material for: Increase of microRNA-210, Decrease of Raptor Gene Expression and Alteration of Mammalian Target of Rapamycin Regulated Proteins following Mithramycin Treatment of Human Erythroid Cells
Source: PLoS One. 2015 Apr 7;10(4):e0121567. doi: 10.1371/journal.pone.0121567 (PMC4388523; doi:10.1371/journal.pone.0121567)
Supplement: S3 Table — (DOC) [file pone.0121567.s004.doc]

| **Pathway** | **Number of Entities** | **Matched with Technology** | **Matched with Entity List** | **p-value** |
| --- | --- | --- | --- | --- |
| hemoglobins chaperone | 25 | 12 | 6 | 0.0000000062 |
| Fc-epsilon receptor I signalling in mast cells | 97 | 49 | 6 | 0.0009437549 |
| TCR | 140 | 125 | 10 | 0.0016823744 |
| Neurophilin interactions with VEGF and VEGFR | 6 | 3 | 2 | 0.0020193104 |
| BH3-only proteins associate with and inactivate anti-apoptotic BCL-2 members | 9 | 3 | 2 | 0.0020193104 |
| IL3 | 76 | 71 | 7 | 0.0025445842 |
| BCR | 148 | 133 | 10 | 0.0026612065 |
| Sphingosine 1-phosphate (S1P) pathway | 223 | 106 | 8 | 0.0027947320 |
| Syndecan-2-mediated signaling events | 131 | 44 | 5 | 0.0033062470 |
| ionomycin and phorbal ester signaling pathway | 12 | 4 | 2 | 0.0044284454 |
| FGF signaling pathway | 78 | 30 | 4 | 0.0052984900 |
| IL2 signaling events mediated by PI3K | 110 | 33 | 4 | 0.0059043947 |
| Ceramide signaling pathway | 90 | 55 | 5 | 0.0075754270 |
| TNF receptor signaling pathway | 495 | 213 | 11 | 0.0090316170 |
| IL2-mediated signaling events | 184 | 64 | 5 | 0.0138245640 |
| Visual signal transduction: Cones | 51 | 9 | 2 | 0.0150567540 |
| Visual signal transduction: Rods | 50 | 11 | 2 | 0.0223588300 |
| Proteogylcan syndecan-mediated signaling events | 525 | 187 | 9 | 0.0244462080 |
| S1P5 pathway | 7 | 1 | 1 | 0.0261977580 |
| Activation of PUMA and translocation to mitochondria | 1 | 1 | 1 | 0.0261977580 |
| RNA Polymerase I Promoter Opening | 4 | 1 | 1 | 0.0261977580 |
| telomeres telomerase cellular aging and immortality | 28 | 11 | 2 | 0.0264530870 |
| Intrinsic Pathway for Apoptosis | 32 | 12 | 2 | 0.0264530870 |
| mechanism of gene regulation by peroxisome proliferators via ppara | 22 | 10 | 2 | 0.0268167800 |
| Signaling events mediated by VEGFR1 and VEGFR2 | 141 | 51 | 4 | 0.0288069080 |
| regulation of bad phosphorylation | 30 | 13 | 2 | 0.0354568920 |
| erk and pi-3 kinase are necessary for collagen binding in corneal epithelia | 24 | 13 | 2 | 0.0354568920 |
| Aminosugars metabolism | 71 | 32 | 3 | 0.0362297700 |
| EPO signaling pathway | 64 | 37 | 3 | 0.0390118100 |
| FOXA2 and FOXA3 transcription factor networks | 56 | 50 | 4 | 0.0416051750 |
| Canonical NF-kappaB pathway | 65 | 15 | 2 | 0.0454583760 |
| Integrins in angiogenesis | 107 | 38 | 3 | 0.0479856000 |
